# Oxidative Stress Induced by Antivirals: Implications for Adverse Outcomes During Pregnancy and in Newborns

**DOI:** 10.3390/antiox13121518

**Published:** 2024-12-12

**Authors:** Bárbara Costa, Maria João Gouveia, Nuno Vale

**Affiliations:** 1 PerMed Research Group, Center for Health Technology and Services Research (CINTESIS), 4200-450 Porto, Portugal; bcosta@med.up.pt; 2 CINTESIS@RISE, Faculty of Medicine, University of Porto, 4200-319 Porto, Portugal; 3 Department of Community Medicine, Health Information and Decision (MEDCIDS), Faculty of Medicine, University of Porto, 4200-319 Porto, Portugal; 4 Centre for Parasite Biology and Immunology, Department of Infectious Diseases, National Health Institute Dr. Ricardo Jorge, 4000-055 Porto, Portugal; m.joao.gouveia@insa.min-saude.pt; 5 Center for the Study in Animal Science (CECA/ICETA), University of Porto, 4051-401 Porto, Portugal

**Keywords:** oxidative stress, antivirals, pregnancy, adverse outcomes, pharmacokinetics, in vitro studies, therapeutic strategies, drug safety, maternal health, fetal development

## Abstract

Oxidative stress plays a critical role in various physiological and pathological processes, particularly during pregnancy, where it can significantly affect maternal and fetal health. In the context of viral infections, such as those caused by Human Immunodeficiency Virus (HIV) and severe acute respiratory syndrome coronavirus 2 (SARS-CoV-2), oxidative stress may exacerbate complications by disrupting cellular function and immune responses. Antiviral drugs, while essential in managing these infections, can also contribute to oxidative stress, potentially impacting both the mother and the developing fetus. Understanding the mechanisms by which antivirals can contribute to oxidative stress and examination of pharmacokinetic changes during pregnancy that influence drug metabolism is essential. Some research indicates that antiretroviral drugs can induce oxidative stress and mitochondrial dysfunction during pregnancy, while other studies suggest that their use is generally safe. Therefore, concerns about long-term health effects persist. This review delves into the complex interplay between oxidative stress, antioxidant defenses, and antiviral therapies, focusing on strategies to mitigate potential oxidative damage. By addressing gaps in our understanding, we highlight the importance of balancing antiviral efficacy with the risks of oxidative stress. Moreover, we advocate for further research to develop safer, more effective therapeutic approaches during pregnancy. Understanding these dynamics is essential for optimizing health outcomes for both mother and fetus in the context of viral infections during pregnancy.

## 1. Introduction

Antiviral medications are often prescribed during pregnancy to manage viral infections that could pose significant risks to the mother and fetus. As remarked by Money et al., these therapies are more recent compared to traditional antibiotics, particularly in the context of pregnancy, where long-term safety data are sparse [1]. This lack of data is partly due to the fact that during pregnancy, individuals are often excluded from clinical trials, leaving a gap in understanding regarding the full effects of these medications during pregnancy [2]. As a result, several novel drugs may not be recommended for use in pregnant individuals since their safety has not been adequately studied in this population [3]. When considering treatment options, it remains important to prioritize up-to-date information to ensure safe care for the mother and the fetus. Avoiding unnecessary medications during the first trimester is still advisable, and careful consideration and judicious use of these therapies in later pregnancy is essential. The potential risks of inadvertent drug exposure due to unplanned pregnancies must be weighed against the need for effective treatment, particularly given the uncertainties surrounding newer medications [4]. Drug exposure affects the fetus differently depending on gestational age, with examples including nonsteroidal anti-inflammatory drugs (NSAIDs) causing ductus arteriosus constriction in early pregnancy, teratogenic agents leading to malformations during organogenesis, beta-2 agonists [5] and selective serotonin reuptake inhibitors (SSRIs) influencing fetal heart function in later stages [6], and corticosteroids and warfarin [7] having varying impacts depending on the timing of exposure [8,9]. Determining safe treatments requires balancing medical necessity with the risk of inadvertent early exposure due to unplanned pregnancies. This complexity is intensified by the potential for antivirals to induce oxidative stress, which can have harmful effects.

**Figure 1:**
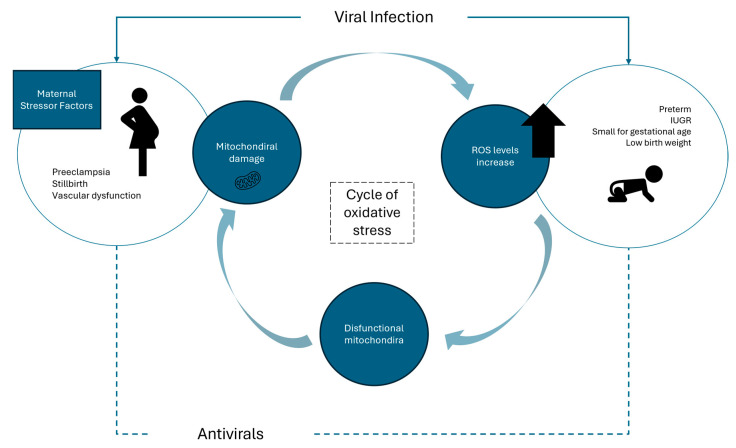
Oxidative stress is a mechanism for adverse outcomes for pregnant and newborns. Viral infections and the use of antivirals/antiretrovirals are associated with oxidative stress, which can have significant implications for maternal and neonatal health (e.g., intrauterine growth restriction (IUGR)). Image adapted from Nüsken et al. [20].

Oxidative stress occurs when there is an imbalance between reactive oxygen species (ROS) and the body’s capacity to neutralize them, leading to cellular and tissue damage. During pregnancy, this can impact both maternal and fetal health, potentially resulting in complications such as preeclampsia, intrauterine growth restriction, and preterm labor [10,11]. Pregnancy itself is associated with increased oxidative stress due to heightened metabolic demands and physiological changes. The placenta, which plays an essential role in the signaling and regulation of cellular processes, is a major source of ROS [12]. Viral infections can exacerbate oxidative stress during pregnancy (Figure 1). For example, severe acute respiratory syndrome coronavirus 2 (SARS-CoV-2) infection triggers a systemic inflammatory response, which can release pro-inflammatory cytokines [13], leading to uncontrolled infection, lymphocyte depletion, and increased tissue damage [11]. This inflammatory state is associated with increased oxidative stress, as inflammation can enhance the production of ROS. Preliminary findings by Mandò et al. suggest that the placentas of mothers infected with SARS-CoV-2 exhibit reduced levels of mitochondrial DNA (mtDNA) and altered expression of genes involved in mitochondrial function, particularly those related to mitochondrial dynamics and respiratory chain activity. Mitochondrial dysfunction in these placentas can impair cellular energy production and increase ROS generation, further exacerbating oxidative stress [14]. The body’s defense against oxidative stress relies on a complex network of enzymatic antioxidants. Key enzymes such as superoxide dismutase (SOD), catalase (CAT), and glutathione peroxidase (GPx) play critical roles in neutralizing ROS. For instance, SOD catalyzes the dismutation of superoxide radicals, while CAT breaks down hydrogen peroxide into water and oxygen [15]. During pregnancy, these enzymatic mechanisms become even more crucial. Peroxiredoxins and thioredoxin reductase contribute additional layers of cellular protection, helping to mitigate the increased oxidative stress associated with physiological changes and potential viral infections like SARS-CoV-2 [16]. The reduced expression of antioxidant defense genes, such as CAT and GSS, as observed in SARS-CoV-2-infected placentas further underscores the importance of these enzymatic systems in maintaining cellular integrity during pregnancy. This reduction compromises the placenta’s ability to neutralize ROS, intensifying oxidative stress. The acute effects of viral infection, coupled with potential oxygen desaturation and placental malperfusion, may hinder the placenta’s ability to activate compensatory mechanisms normally employed to mitigate oxidative damage [14]. There is evidence that the placenta plays a protective role in preventing fetal infection with SARS-CoV-2, and recent research has indicated that SARS-CoV-2 infection during the third trimester does not necessarily lead to significant changes in placental histology compared to controls, suggesting that the timing and duration of exposure may influence the placenta’s response [17,18]. While there is growing evidence linking SARS-CoV-2 infection to oxidative stress and adverse pregnancy outcomes, including impacts on both the mother and neonate, this relationship is still being actively researched. The exact mechanisms by which SARS-CoV-2-induced oxidative stress contributes to these outcomes remain unclear, and more studies are needed to fully understand the interplay among viral infection, oxidative stress, and placental function during pregnancy.

Given the widespread use of antivirals during pregnancy, it is crucial to explore how these drugs might induce oxidative stress and the implications for maternal and neonatal health. Antiviral drugs are crucial for managing viral infections, yet their effects on oxidative balance in pregnancy are not fully understood. Some antiviral medications may contribute to oxidative stress, potentially exacerbating the already elevated levels associated with pregnancy and viral infections [19]. This review aims to (i) provide a comprehensive analysis of the mechanisms by which antivirals may trigger oxidative stress, (ii) evaluate the potential risks associated with their use during pregnancy, and (iii) highlight the need for further research to ensure the safety of mothers and their newborns.

## 2. Viral Infections and Their Role in Oxidative Stress

Virus-induced toxicity involves complex mechanisms that lead to cellular damage and tissue dysfunction, with oxidative stress playing a central role. Understanding these mechanisms is crucial for developing targeted antiviral therapies and improving treatment outcomes. During viral replication, ROS are produced, causing oxidative damage to lipids, proteins, and DNA, which can result in cellular dysfunction or death. Although host cells activate antioxidative defense systems to restore redox balance, excessive ROS generated by viral activity and immune responses can result in tissue damage and inflammation. Viruses can alter host metabolism and modulate cellular processes, contributing to toxicity by manipulating mitochondrial functions to increase ROS levels [21,22]. Paradoxically, this can support viral replication while simultaneously triggering host antiviral responses. This interaction may also disrupt mitochondrial dynamics, such as morphology and membrane potential, which are critical for ROS production [23]. For instance, SARS-CoV-2 has been shown to manipulate mitochondrial functions to increase ROS levels. This manipulation helps the virus replicate more efficiently while triggering the host’s antiviral responses [19]. This increased ROS production contributes to the severe inflammatory responses seen in COVID-19 patients, often leading to acute respiratory distress syndrome (ARDS) and other complications [24]. Hepatitis C virus (HCV) is another example where viral manipulation of mitochondrial functions plays a crucial role [25]. HCV infection leads to increased ROS production, which can cause liver inflammation and fibrosis [26]. The virus alters mitochondrial dynamics and disrupts the electron transport chain, leading to increased production of ROS. These ROS, along with pro-inflammatory cytokines, activate hepatic stellate cells, which play a crucial role in liver fibrosis [27]. Once activated, hematopoietic stem cells (HSCs) transform into myofibroblast-like cells that produce extracellular matrix (ECM) components, leading to fibrosis. After that, an inflammatory response is triggered, releasing cytokines such as tumor necrosis factor (TNF)-α, interleukin-1 (IL-1), and transforming growth factor beta (TGF-β). These cytokines further enhance ROS production and perpetuate the cycle of inflammation and fibrosis [28,29]. Interestingly, the oxidative environment created by ROS also supports HCV replication. The virus benefits from oxidative stress to maintain its lifecycle, thereby sustaining chronic infection and ongoing liver damage [30].

The influenza virus induces inflammatory responses through several mechanistic pathways, primarily by increasing intracellular ROS levels, which disrupt the redox balance in host cells. During infection, ROS are generated as byproducts of mitochondrial metabolism, and elevated ROS production leads to oxidative stress, causing cellular damage. This imbalance promotes viral replication by compromising the host’s immune defenses and enhancing viral entry and replication within cells. Additionally, the accumulation of ROS triggers programmed cell death (apoptosis) and stimulates the release of proinflammatory cytokines and chemokines, such as interferons (IFNs), TNFs, and interleukins (ILs) [31]. This cytokine storm exacerbates tissue damage, particularly in the lungs, leading to severe respiratory symptoms and contributing to the pathogenesis of influenza. The virus also hijacks host cell signaling pathways, such as the JNK/ERK/p38 MAPK and NF-κB pathways, which are activated by ROS, amplifying inflammation and lung injury [31,32]. Thus, the interplay among viral replication, oxidative stress, and the immune response creates a cycle in which ROS-induced tissue damage both facilitates viral replication and triggers a harmful inflammatory response, hindering the host’s recovery without intervention. Not surprisingly, human immunodeficiency virus (HIV) infection is also associated with increased oxidative stress due to the virus’s ability to manipulate host cell signaling pathways. The virus activates NADPH oxidase, leading to elevated ROS levels [33], contributing to the chronic inflammation and immune activation seen in HIV-infected individuals. Additionally, increased ROS levels can damage several tissues conducive to the development of comorbidities such as cardiovascular disease [34].

There are several cellular defense mechanisms against oxidative stress, including the nuclear factor erythroid 2-related factor 2 (Nrf2) pathway. Under normal conditions, Nrf2 is kept in the cytoplasm and degraded [35]. However, in response to oxidative stress, Nrf2 is released and translocated to the nucleus, activating the transcription of various antioxidant genes. These genes encode proteins that help detoxify ROS and protect cells from oxidative damage. Thus, the Nrf2 pathway plays a vital role in enhancing the host’s antioxidative response to counteract virus-induced oxidative stress [36]. Interestingly, viruses can exploit this pathway to their benefit. Some viruses can enhance the Nrf2 pathway to promote their replication (positive modulation). For instance, certain viral proteins can induce oxidative stress that activates Nrf2, leading to the increased expression of antioxidant genes that may support viral metabolism. Conversely, other viruses may suppress the Nrf2 pathway to evade the host’s antioxidative defenses (negative modulation) [37,38]. HBV and HCV can disrupt Nrf2 signaling pathways, contributing to liver damage and promoting viral replication [39]. Influenza A virus can also interfere with Nrf2 signaling [40]. SARS-CoV-2 has been shown to dysregulate Nrf2 activity, which may contribute to the severe inflammatory responses seen in some patients [41]. The modulation of the Nrf2 pathway by viruses has significant implications for the progression of viral diseases [42]. When viruses positively regulate the Nrf2 pathway, it may help them thrive by creating a favorable environment for replication. On the other hand, if a virus suppresses the Nrf2 pathway, it can lead to uncontrolled oxidative stress, resulting in cell death and exacerbating the disease [43].

During pregnancy, three major factors render viral infections particularly concerning. First, altered immune response: pregnancy induces changes in the immune system that may affect the response to viral infections. A significantly attenuated interferon (IFN) response exists in isolated peripheral mononuclear cells [44]. The adaptive immune responses may be altered, with some studies showing diminished induction of certain antibody responses during the later stages of pregnancy. Second, increased susceptibility and physiological adaptations during pregnancy, including immunological and endocrinological alterations, render pregnant individuals susceptible to certain viral and bacterial infections. Third, viral infections during pregnancy pose risks to the mother and the developing fetus. This includes risks of maternal morbidity, pregnancy loss, stillbirth, intrauterine growth restriction, preterm birth, neonatal death, and congenital abnormalities [45]. At last, it is necessary to consider placental involvement; the placenta can be affected by viral infections [46]. Viruses can replicate in placental cells, potentially disrupting placental function and increasing the risk of vertical transmission [47]. The interplay between viral infections, the altered immune response during pregnancy, and oxidative stress creates a complex environment that can significantly impact maternal and fetal health. Understanding these interactions is crucial for developing effective strategies to prevent and safely manage viral infections during pregnancy [48]. Future research should focus on elucidating the specific mechanisms by which different viruses interact with the maternal–fetal unit and how oxidative stress modulates these interactions.

During pregnancy, the Nrf2 pathway also plays a dual role in viral infections, offering protective and potentially harmful effects. The placenta, which is particularly vulnerable to oxidative stress during viral infections, may benefit from Nrf2 activation in placental cells, helping to protect against virus-induced damage and maintain placental function [49,50]. Nrf2 plays a crucial role in fetal development by protecting against oxidative stress; modulating its activity could potentially reduce the impact of viral infections on the fetus [51]. Therapeutic strategies targeting the Nrf2 pathway are currently being explored. Selective Nrf2 activators are being developed to enhance antioxidant defenses against virus-induced oxidative stress without promoting viral replication, but these compounds must be carefully designed to avoid negative effects on fetal development [52]. Similarly, delivery systems targeted to the infected tissues, or the placenta could maximize benefits while minimizing systemic effects [53]. The timing of intervention, influenced by the stage of pregnancy and timing of infection, is critical for ensuring the safety and effectiveness of Nrf2-targeted therapies. Considering individual genetic variability and specific viral strains, personalized approaches may be necessary. Understanding these dynamics is crucial for developing therapeutic strategies that target pathways to enhance the host’s antioxidative response and mitigate viral pathogenesis.

## 3. The Link Between Oxidative Stress and Antiviral Agents

### 3.1. Impact of Antiviral Medications on Antioxidant Mechanisms

Oxidative stress arises from several mechanisms, such as mitochondrial dysfunction [54], glutathione depletion, lipid peroxidation, and inflammation. Many antivirals, particularly transcriptase inhibitors (NRTIs), disrupt mitochondrial function, increasing ROS production. Despite being effective in controlling viral replication, some antivirals can paradoxically amplify inflammatory responses, indirectly contributing to oxidative stress [55]. The effects of antivirals on oxidative stress are particularly concerning during pregnancy, as they can contribute to adverse outcomes like fetal growth restriction and preterm birth. Several approaches are being explored to address these major concerns: (1) Antioxidant supplementation and co-administration of antioxidants with antiviral therapy to counteract oxidative stress [56]. (2) Developing less toxic antivirals focused on maintaining efficacy while reducing their impact on cellular antioxidant systems [57]. However, pregnant individuals often lack access to these novel drugs due to limited safety data; there has not been enough time to fully assess their long-term effects during pregnancy [58]. (3) Personalized medicine: tailoring antiviral regimens based on individual oxidative stress profiles and genetic factors can also produce better outcomes [59,60]. More research is needed to ensure their safe use in this population.

Common antiviral medications include NRTIs, protease inhibitors, and neuraminidase inhibitors. NRTIs, like zidovudine and lamivudine, inhibit viral replication by incorporating themselves into viral DNA, causing chain termination [22]. Protease inhibitors, such as ritonavir, block the protease enzyme, which is essential for viral maturation [23], while neuraminidase inhibitors, like oseltamivir, prevent the release of new viral particles from infected cells [24]. While these drugs are effective in controlling viral infections, their mechanisms can inadvertently contribute to oxidative stress. Research has shown that antiretroviral therapies (ARVs), particularly protease inhibitors like ritonavir and saquinavir, can induce oxidative stress and neuronal damage, especially in the central nervous system. Studies demonstrated that ARVs can cause synaptic injury and neuronal death, with evidence of oxidative stress markers persisting in the brains of patients on combination antiretroviral therapy (cART), even when viral replication is controlled. This suggests that ARVs control infections but may also contribute to neuronal damage through oxidative stress mechanisms [25].

Zidovudine and lamivudine have been associated with mitochondrial toxicity, leading to increased ROS production due to impaired electron transport chain function [26]. This can result in oxidative damage to mitochondrial DNA, proteins, and lipids, which may exacerbate cellular dysfunction [27]. The drug efavirenz, which inhibits reverse transcriptase by binding to a site distinct from NRTIs [28], has been associated with liver damage (hepatotoxicity) that may be partly caused by oxidative stress mechanisms [29]. Protease inhibitors can disrupt lipid metabolism, increasing free fatty acid levels and oxidative stress and potentially inducing endoplasmic reticulum (ER) stress, triggering the unfolded protein response and increasing ROS production [30,31]. These oxidative effects are particularly concerning during pregnancy, as they could possibly affect fetal development and maternal health [32]. Neuraminidase inhibitors like oseltamivir, primarily used against influenza viruses, prevent the release of newly formed viral particles from infected cells [33]. Generally, they have fewer oxidative stress-related side effects compared to NRTIs, and protease inhibitors have suggested potential oxidative effects. Evidence indicates that neuraminidase inhibitors may influence cellular redox signaling pathways, potentially affecting oxidative stress levels [34,35]. Integrase inhibitors, generally well-tolerated, have been reported to cause mitochondrial dysfunction. Interestingly, raltegravir and dolutegravir increase oxidative stress and mitochondrial dysfunction while simultaneously promoting lipogenesis and adipogenesis [36]. Moreover, it has been demonstrated that dolutegravir increases cellular mass, mitochondrial ROS, and apoptosis and disrupts mitochondrial networks in human fibroblast WI-38. The effects of integrase strand transfer inhibitors are still not fully understood, and additional research in cohorts with female participants to support the action of hormones on mitochondria is necessary to determine whether there are any alterations in mtDNA or biogenesis [37].

Antivirals used for COVID-19 and other viral infections have demonstrated variable effects on oxidative stress [61]. Remdesivir is demonstrated to be effective against SARS-CoV-2; however, studies indicate that it may increase oxidative stress in liver cells, raising concerns about potential hepatotoxicity [62,63]. Similarly, ganciclovir, used in the treatment of cytomegalovirus (CMV) infections, has been shown to induce oxidative stress in certain cell types, potentially contributing to its toxicity profile [61]. Its prodrug, valganciclovir, may exhibit comparable oxidative effects due to its conversion to ganciclovir in vivo. In contrast, tenofovir, widely used for hepatitis B management, is generally well-tolerated, with limited direct evidence suggesting a significant impact on oxidative stress. This positions tenofovir as a relatively safer antiviral option in terms of oxidative balance [64,65]. Direct-acting antivirals for Hepatitis C, while highly effective in achieving viral clearance, have been reported to temporarily increase oxidative stress markers during treatment in some studies [62]. This transient oxidative stress may not have long-term adverse effects but warrants consideration in specific patient populations [66]. Finally, protease inhibitors are associated with increased oxidative stress, which contributes to metabolic complications such as insulin resistance and dyslipidemia [63]. These findings underscore the need for the careful monitoring of oxidative stress and metabolic health in patients receiving protease inhibitor-based therapies [63]. These findings underscore the need for careful monitoring of oxidative stress and metabolic health in patients receiving protease inhibitor-based therapies [67].

Antiviral drugs can contribute to oxidative stress and, by contrast, might have minimal impact on oxidative stress or even possess antioxidant properties. For example, umifenovir (arbidol), an antiviral drug used against influenza, has demonstrated antioxidant potential through the inhibition of lipid peroxidation and the prevention oxidative damage to the mitochondrial respiratory chain [68]. The drug exhibits antiviral and antioxidant effects, potentially protecting cells from oxidative stress induced by viral infections. These features might influence cellular redox signaling pathways, diminishing their impact on oxidative stress in comparison with other NRTIs or protease inhibitors. Similarly, silymarin, echinichrom A, and arctigenin also possess both activities. Silymarin has shown potent antiviral activity against the Mayaro virus and antioxidant activity by reducing biomarkers of oxidative stress [69]. Echinochrome A, when combined with ascorbic acid and alpha-tocopherol, demonstrated higher antioxidant and antiviral effects than echinochrome A alone [70]. Arctigenin has shown both antioxidant and antiviral activities against Japanese encephalitis virus (JEV) infection [71].

To address oxidative stress concerns with antiviral therapy, it is possible to (a) combine drugs with antioxidants to counteract oxidative effects. For example, interferon alpha-2b combined with unithiol showed synergistic antiviral effects against Herpes simplex virus [72]; (b) develop dual-action drugs creating antiviral compounds with inherent antioxidant properties; (c) administer antioxidants alongside antiviral therapy to mitigate oxidative stress or even consider non-thermal plasma (NTP) therapy. This emerging approach manipulates oxidative stress responses to treat viral infections like herpes simplex virus type 1, potentially offering an alternative to traditional antiviral drugs [73].

Antiviral medications are essential for managing viral infections; nevertheless, a deeper understanding of their interference with antioxidant mechanisms is required. This will provide better monitoring and the development of supportive strategies to minimize adverse effects, especially in vulnerable populations like pregnant. Most studies have focused on in vitro or animal models, with limited data available from human studies. There is a lack of comprehensive studies evaluating the long-term effects of antiviral-induced oxidative stress on both maternal and neonatal outcomes. Additionally, the potential for antioxidant therapies to mitigate these effects has not been adequately explored. Addressing these gaps is essential for developing safer antiviral treatment protocols during pregnancy and reducing the risk of adverse outcomes for both mothers and their newborns.

### 3.2. Challenges in Assessing Oxidative Stress Caused by Antivirals

Assessing oxidative stress caused by antivirals presents several challenges, including the complexity of accurately measuring ROS and their impact on biological systems. For example, ROS levels fluctuate rapidly, making it difficult to capture real-time oxidative stress [74]. Furthermore, the effects of antivirals on oxidative stress vary based on the drug class, dosage, duration of treatment, and individual patient factors such as genetics and underlying health conditions. Another challenge is distinguishing oxidative stress directly induced by the antiviral itself from viral infection or inflammation effects. The lack of standardized biomarkers to reliably assess oxidative stress in clinical settings complicates the evaluation of its long-term impact, especially in sensitive populations like pregnancy, where oxidative stress can contribute to adverse outcomes. Finally, the interplay between antiviral efficacy and oxidative damage increases complexity, as drugs designed to control viral replication may inadvertently exacerbate oxidative stress [75]. Table 1 summarizes the challenges of assessing oxidative stress with antivirals. Usually, the body maintains a balance between ROS production and antioxidant activity through endogenous systems like SOD, CAT, and GPx. However, when this balance is disrupted, oxidative stress occurs, which can lead to inflammation, apoptosis, and tissue damage [76]. Since antivirals can induce ROS production through different mechanisms, it is challenging to pinpoint the exact source [77].

## 4. Impact of Antivirals During Pregnancy and Adverse Outcomes in Newborns

### 4.1. Oxidative Balance During Pregnancy and Viral Infection

Oxidative stress is a physiological phenomenon during pregnancy, primarily arising from increased placental metabolism and oxygen demand. While oxidative stress plays a critical role in cellular signaling and fetal development, its effects are not exclusively harmful. Under normal conditions, the body’s antioxidant defenses maintain a balance, allowing oxidative stress to support essential physiological processes. However, when this balance is disrupted and oxidative stress becomes excessive, it can lead to complications such as preeclampsia, IUGR, and preterm birth. Understanding this dual role is essential for evaluating the impact of external factors, such as viral infections and antiviral therapies, on maternal and fetal health [78].

Viral infections, such as SARS-CoV-2, can disrupt this balance, amplifying oxidative stress and inflammation and impair placental function [79]. The placenta, a critical organ in regulating maternal–fetal exchange and immune responses, is highly vulnerable to oxidative stress. In pregnant women infected with SARS-CoV-2, mitochondrial and oxidative imbalances in the placenta have been linked to adverse outcomes, such as placental dysfunction and impaired fetal development [14]. Research has shown that placental Nrf2 levels decrease in pregnant COVID-19-positive patients, especially those with symptomatic infections, and are associated with increased oxidative damage [80]. This mitochondrial dysfunction disrupts the placenta’s ability to manage ROS, leading to heightened oxidative stress. The expression of key antioxidant genes and mitochondrial respiratory chain components is also reduced in COVID-19-affected placentas, further compounding oxidative damage [14,79]. The placental oxidative stress during pregnancy with COVID-19 may contribute to systemic endothelial dysfunction, impaired placental angiogenesis, and compromised transport mechanisms. These alterations can result in pregnancy complications such as preeclampsia, intrauterine growth restriction, and fetal developmental issues [81]. Iron metabolism is also significantly altered in these cases, with increased iron uptake and storage in the placenta contributing to oxidative stress through the Fenton and Haber–Weiss reactions [82]. Moreno-Fernandez et al. highlights an adaptive increase in certain antioxidant vitamins, such as vitamin D, E, and coenzyme Q10, in the placentas of COVID-19 patients. This increase is hypothesized as a compensatory mechanism to mitigate the oxidative stress induced by the viral infection. However, despite these adaptive responses, the overall oxidative imbalance can still have long-term effects on maternal and fetal health [79]. The findings emphasize the importance of understanding how viral infections, like SARS-CoV-2, affect the oxidative balance during pregnancy, as this can lead to immediate and long-term adverse outcomes for the newborn. Addressing this oxidative stress through appropriate interventions may be crucial in reducing the risk of pregnancy complications and improving fetal development.

Several viruses and consequent infections can affect oxidative balance during pregnancy in distinct ways, which may impact fetal growth and development. For instance, Influenza A virus (IAV) infection during pregnancy leads to the enhanced production of ROS by activated immune cells like macrophages and neutrophils and the depletion of antioxidant defenses, including enzymes and non-enzymatic antioxidants [83], triggering an exacerbated inflammatory response contributing to oxidative stress. The oxidative imbalance might lead to several maternal complications, such as (a) acute cardiopulmonary distress syndrome, increasing the risk of severe illness and hospital intensive care unit (ICU) admission; (b) vascular dysfunction that potentially contributes to a “Vascular Storm” that resembles preeclampsia [84]; (c) increased risk of secondary bacterial and viral pneumonia with higher morbidity and mortality compared to non-pregnant patients [85].

IAV infection-induced oxidative stress is associated with adverse pregnancy outcomes, namely, a higher risk of stillbirth, particularly during pandemic influenza seasons. Additionally, there is a potential increased risk of preterm birth (especially when fever symptoms exist) and reduced birth weight in full-term newborns. The impact of IAV infection on oxidative stress and pregnancy outcomes is variable based on the timing of infection, with the highest incidence occurring during the first trimester. The major severity cases of the infection are associated with greater oxidative imbalance and worse outcomes [83]. In the case of HIV, the oxidative imbalance can increase the risk of opportunistic infections due to compromised immune function, potential exacerbation of HIV-related symptoms and disease progression, and higher susceptibility to pregnancy-related complications like preeclampsia. Studies have shown specific redox imbalance markers in HIV-infected pregnant patients [86]. Higher levels of malondialdehyde (MDA) indicate increased lipid peroxidation. Elevated activity of antioxidant enzymes like SOD and CAT suggests a compensatory response to increased oxidative stress. Changes in protein carbonylation levels indicate protein oxidation. Understanding the relationship between HIV infection, pregnancy, and oxidative stress has important implications, highlighting the need for close monitoring of HIV-infected pregnant patients for signs of oxidative stress-related complications [87].

HBV and HCV infections are responsible for an increased risk of gestational diabetes, particularly with HBV infection [88,89]. Higher susceptibility to pregnancy-related complications like preeclampsia, specifically HBV infection, is associated with a 10% increased risk of preeclampsia compared to uninfected individuals [90]. There is also a potential exacerbation of liver disease, especially in women with pre-existing chronic hepatitis. HBV and HCV infections can increase the susceptibility of placental cells to apoptosis and alter placental barrier function, potentially increasing the risk of vertical transmission [91]. Disruption of normal placental development and function may contribute to adverse pregnancy outcomes [92]. Oxidative stress from HBV and HCV infections during pregnancy may have long-term health implications for the offspring, including the potential increased risk of chronic liver disease in children infected perinatally and possible developmental issues related to prenatal exposure to oxidative stress [93].

CMV infection can disrupt placental function by increasing the susceptibility of syncytiotrophoblast cells to apoptosis [94,95]. CMV infection induces chronic lymphoplasmacytic villitis, characterized by inflammation of the placental villi. This inflammation can impair the placenta’s ability to support the fetus [96,97]. CMV infection leads to increased levels of pro-inflammatory cytokines such as MCP-1 (CCL2) and TNF-α in the placenta. These cytokines can disrupt normal placental function and contribute to adverse pregnancy outcomes [98]. This virus can directly infect placental cells, causing cytopathic effects such as cell enlargement (cytomegaly) and the formation of intranuclear and intracytoplasmic inclusions; the direct damage can impair placental structure and function [97]. CMV infection can affect the function of cytotrophoblasts, which are crucial for the formation of anchoring villi that attach the placenta to the uterine wall. This impairment can reduce the size and function of these villi, compromising placental attachment and nutrient exchange [99]. These histopathological changes and elevated levels of MCP-1 and TNF-α in the placenta are indicative of significant placental damage and dysfunction and are associated with adverse pregnancy outcomes such as fetal growth restriction and preterm birth. Ex vivo studies using placental explants have shown that CMV infection can significantly increase pro-inflammatory cytokine expression, further supporting cytokine dysregulation’s role in CMV-induced placental dysfunction [99].

Understanding the relationship between viral infections and oxidative stress during pregnancy is crucial for developing targeted interventions. Ongoing research into antioxidant therapies, immune modulation, and tailored care aims to mitigate viral-induced oxidative stress. Balancing the immune response and reducing inflammation could help protect against oxidative damage. Since each virus disrupts oxidative balance differently, strategies such as strict control of oxygen administration, use of antioxidants like lutein and melatonin, and hypothermia to mitigate these risks show promise as potential neuroprotective interventions [100,101]. Further research is essential to prevent and manage these complications effectively.

### 4.2. Pre-Existing Maternal Health Conditions and Antiviral Therapy

Maternal pre-existing conditions, such as diabetes, hypertension, obesity, and chronic viral infections, can amplify the oxidative stress burden during pregnancy, creating a complex interplay between maternal health and antiviral therapy. These conditions are often associated with elevated baseline levels of ROS and reduced antioxidant defenses, increasing susceptibility to oxidative damage [102].

Antiviral drugs, while essential for managing maternal viral infections, may further exacerbate oxidative stress. Increased oxidative stress can worsen glycemic control, leading to further complications such as gestational diabetes or preeclampsia. The accumulation of free radicals from antiviral therapy may impair vascular function, increasing the risk of placental dysfunction and preeclampsia. Obesity-related oxidative stress, combined with drug-induced ROS production, could heighten systemic inflammation, impacting both maternal and fetal health [103].

The combined effect of pre-existing conditions and antiviral therapy-induced oxidative stress may accelerate disease progression. In mothers with chronic viral infections such as HIV or hepatitis, antivirals like NRTIs are known to cause mitochondrial dysfunction, which can worsen oxidative stress and heighten the risk of liver damage or cardiovascular disease. Chronic inflammation in these conditions, compounded by drug-induced ROS, could increase the risk of adverse outcomes such as preterm labor or fetal growth restriction [104].

Addressing oxidative stress in mothers with pre-existing conditions requires tailored therapeutic strategies. These may include the co-administration of antioxidants with antiviral therapy or the selection of antivirals with minimal oxidative effects. Personalized treatment plans based on the mother’s health status and oxidative stress biomarkers can help mitigate risks, ensuring better maternal and fetal outcomes.

### 4.3. Adverse Outcomes in Newborns Caused by Viral and Antiviral-Induced Oxidative Stress

Oxidative stress, resulting from an imbalance between pro-oxidant and antioxidant factors, poses significant risks to newborns, especially preterm infants [105]. At birth, newborns experience a sudden increase in oxygen availability, leading to enhanced free radical generation [106]. Preterm infants are particularly vulnerable due to immature antioxidant systems and reduced ability to control free radical overproduction [107]. Oxidative stress is implicated in various neonatal conditions, including bronchopulmonary dysplasia, retinopathy of prematurity, and intraventricular hemorrhage [108]. Oxidative stress is linked to respiratory conditions like bronchopulmonary dysplasia (BPD) and respiratory distress syndrome (RDS) in preterm infants. It can also contribute to brain injuries, potentially leading to long-term neurodevelopmental issues. Oxidative stress can exacerbate inflammation, increasing newborns’ susceptibility to infections [105,109]. Advanced analytical techniques using mass spectrometry can help detect specific biomarkers for the improved diagnosis and treatment of oxidative stress-related conditions in newborns. The most relevant biomarkers of oxidative stress during the neonatal period include byproducts of oxidative damage to proteins, lipids, and DNA, and they are measurable in fluids like blood, urine, and amniotic fluid (Table 2). Oxidative stress biomarkers are crucial for assessing neonatal health, particularly in preterm infants who are more susceptible to oxidative stress-related complications [110]. Key biomarkers include isoprostanes, advanced oxidation protein products (AOPP), and non-protein-bound iron (NPBI), which reflect lipid, protein, and DNA oxidation, respectively [110]. These markers have shown diagnostic and prognostic value in various neonatal diseases [106,107]. Traditionally, blood samples were used for biomarker analysis, but there is a growing trend toward non-invasive sampling methods to reduce pain and excessive blood extraction in neonatal intensive care units [106]. Cord blood, urine, and saliva have been identified as valid and ethically acceptable biological samples for oxidative stress biomarker analysis in the perinatal period [106,107]. High-performance liquid chromatography–tandem mass spectrometry has been developed to accurately determine these biomarkers in non-invasively obtained biofluids [110]. Key biomarkers include the glutathione/glutathione disulfide (GSH/GSSG) ratio, which reflects the redox status and is a reliable indicator of oxidative stress and it is measured in umbilical cord blood, whole blood, or tissue Biomarkers like the o-Tyr/Phe, m-Tyr/Phe, 3NO_2_-Tyr/p-Tyr, and 3Cl-Tyr/p-Tyr ratios, which indicate protein modifications due to oxidative stress, such as tyrosine nitration and chlorination, detectable in urine, plasma, or milk; the 8-oxodG/2dG ratio (used to detect oxidative DNA damage), commonly assessed in urine or plasma. Finally, biomarkers like isoprostanes (IsoPs), isofurans (IsoFs), and neuroprostanes (NeuroPs) indicate lipid peroxidation. Altogether, these biomarkers are critical for assessing oxidative stress in neonates, particularly in conditions like hypoxic–ischemic encephalopathy [110]. Oxidative stress during pregnancy can impact fetal development, leading to various adverse outcomes, including neural tube defects, neurodevelopmental disorders, and long-term metabolic syndromes. Research demonstrates that oxidative stress contributes to conditions like respiratory distress syndrome, bronchopulmonary dysplasia, necrotizing enterocolitis, and retinopathy of prematurity [111]. Antivirals, although necessary to control viral infections during pregnancy, can inadvertently trigger these oxidative stress pathways, further complicating neonatal outcomes. In Table 3, we summarize the effect of antivirals on oxidative stress.

Influenza can significantly impact fetal development, primarily through oxidative stress, which may impair placental function and restrict nutrient supply, leading to IUGR. Despite the vertical transmission of IAV being rare, highly pathogenic strains like H5N1 may affect the placenta, and even low-pathogenic strains can cause placental apoptosis and replication issues, contributing to impaired function [112]. This placental dysfunction can result in a variety of adverse fetal outcomes, such as cleft lip, cleft palate, and congenital heart defects [113]. Seasonal influenza, for example, has been associated with increased risks of preterm birth, miscarriage, and fetal growth retardation (FGR), although research findings are sometimes inconsistent [114]. Studies indicate that IUGR and FGR are linked to reduced placental blood flow, limiting fetal oxygen and nutrient delivery [115]. Data from the 2009 influenza pandemic suggested increased risks of fetal death, small for gestational age (SGA) births, and respiratory illnesses in infants born to infected mothers [116,117]. In contrast, other studies have found no significant differences in fetal outcomes during influenza season, leading to debate over the direct impact of IAV on fetal growth.

IUGR resulting from placental dysfunction may also increase the risk of neurodevelopmental disorders such as schizophrenia. Placental inflammation and vascular impairment during maternal infections, compounded by conditions like pregnancy-induced hypertension (PIH) and preeclampsia, have been proposed to contribute to neurodevelopmental issues [118,119]. Animal models support this hypothesis, demonstrating behavioral and brain abnormalities similar to schizophrenia or autism in the offspring of IAV-infected mothers [120,121]. Recent systematic reviews have found no conclusive association between maternal IAV infection during pregnancy and schizophrenia, emphasizing the need for further research into the long-term neuropsychiatric impacts of maternal influenza infection [83]. For HIV, oxidative stress can similarly lead to IUGR, affecting placental function and increasing the risk of vertical transmission if left untreated. The disruption of placental development and function and damage to syncytiotrophoblast cells further contribute to complications. Long-term effects on fetal development and health are also a concern. Hepatitis B and C infections exacerbate the risk of preterm birth, with HBV being associated with a 17% higher risk of preterm delivery compared to uninfected individuals [90]. In addition, these infections can impair placental function, leading to IUGR and increasing the risk of vertical transmission, especially if proper immunoprophylaxis is not provided.

Similarly, CMV infection can result in severe developmental issues, including hearing loss, vision problems, and neurological deficits, with long-term consequences for the child’s development and health [122]. CMV infection can disrupt normal placental function by increasing oxidative stress [123]. Elevated oxidative stress can damage placental cells, impairing nutrients and oxygen transfer to the fetus. Adverse neonatal outcomes cause neurodevelopmental issues, including conditions like microcephaly, sensorineural hearing loss, and growth retardation. Oxidative stress can impair fetal growth, leading to IUGR [124]. Newborns with IUGR are smaller than expected for their gestational age and may face long-term health challenges [125]. CMV-induced oxidative stress can also affect the liver and spleen, leading to conditions like hepatosplenomegaly (enlarged liver and spleen), jaundice, elevated liver enzymes [126], and weaken the neonatal immune system, making infants more susceptible to other infections and illnesses [127].

Antiretroviral therapy during pregnancy, particularly containing zidovudine, has been associated with adverse perinatal outcomes, potentially due to mitochondrial toxicity, oxidative stress, and apoptosis in the placenta [128]. Antiretrovirals have also been linked to hepatotoxicity, cardiotoxicity, and nephrotoxicity, potentially through oxidative stress mechanisms [129]. However, antiviral medications for influenza during pregnancy were not associated with increased rates of preterm birth, premature rupture of membranes, or other adverse outcomes in mothers or neonates [130]. For severe neonatal viral infections like herpes simplex virus and cytomegalovirus, nucleoside analogs remain the primary treatment, but they can cause toxicity and drug resistance. There is a concerning lack of new antivirals in development for potentially fatal neonatal viral infections [131]. Neonatal glucocorticoid therapy is effective for treating chronic lung disease in premature infants but can lead to long-term cardiovascular effects due to oxidative stress and reduced nitric oxide bioavailability. Interestingly, combined treatment with antioxidant vitamins prevented these adverse effects, suggesting a potential protective role for antioxidants in neonatal antiviral therapy [132]. This has significant implications for drug development, where the goal is to balance antiviral efficacy by minimizing oxidative stress to reduce adverse outcomes in newborns. To that, it is necessary to assess how antivirals affect mitochondrial function, ROS production, and placental health.

**Table 2:** Oxidative stress biomarkers.

Biomarker	Type of Oxidative Damage	Significance	Sample Type	References
Isoprostanes	Lipid peroxidation	Reflects oxidative damage to lipids; used as a marker of oxidative stress in preterm infants	Cord blood, urine, saliva	[133,134]
Advanced Oxidation Protein Products (AOPP)	Protein oxidation	Measures protein damage caused by ROS; associated with various neonatal diseases	Cord blood, plasma, saliva	[133,135]
Non-Protein-Bound Iron (NPBI)	Iron-mediated oxidative damage	Indicator of free iron availability, which can catalyze ROS production, linked to oxidative damage	Cord blood, urine	[133]
8-Hydroxy-2′-deoxyguanosine (8-OHdG)	DNA oxidation	Marker of oxidative DNA damage; can indicate long-term risks like cancer or neurodevelopmental disorders	Urine, blood	[136]
Malondialdehyde (MDA)	Lipid peroxidation	Byproduct of lipid peroxidation, associated with cellular damage and oxidative stress in newborns	Cord blood, plasma, urine	[134]
Glutathione (GSH)/Glutathione Disulfide (GSSG)	Redox balance marker	Reflects the cellular oxidative stress status by measuring the balance between reduced and oxidized glutathione	Blood, cord blood, saliva	[133]
Total Antioxidant Capacity (TAC)	Antioxidant defense capacity	Assesses the body’s overall ability to neutralize ROS; useful in determining oxidative stress status	Blood, urine, saliva	[135]

Moreover, oxidative stress plays a significant role in neonatal conditions such as jaundice, respiratory distress syndrome (RDS), and brain injury. In the case of neonatal jaundice, oxidative stress exacerbates bilirubin toxicity by elevating ROS levels, which impair the activity of key enzymes involved in bilirubin metabolism, such as UDP-glucuronosyltransferase, hindering bilirubin conjugation and clearance [137]. This is particularly significant in preterm infants whose antioxidant systems are underdeveloped. Unconjugated bilirubin, in the presence of oxidative stress, can cross the blood–brain barrier, increasing the risk of kernicterus and subsequent neurological complications [138]. In respiratory distress syndrome (RDS), oxidative stress is triggered by oxygen therapy and mechanical ventilation, which leads to excessive ROS production. This damages the alveolar epithelial cells and disrupts surfactant production, exacerbating respiratory failure. Furthermore, oxidative stress-induced inflammation activates cytokine release, which worsens lung injury and increases the risk of bronchopulmonary dysplasia (BPD) in preterm infants [139]. Oxidative stress plays a critical role in neonatal brain injury, including periventricular leukomalacia (PVL) and intraventricular hemorrhage (IVH). ROS cause lipid peroxidation and damage to the immature myelin sheath, impairing neural development. Additionally, oxidative stress activates microglia and astrocytes, leading to neuroinflammation and exacerbating brain damage [140]. This is particularly detrimental in preterm infants, where the antioxidant defenses of the brain are insufficient to counteract ROS.

Optimizing drug dosages, frequency, and delivery methods is crucial to reduce oxidative stress while maintaining effective treatment levels. For example, lowering the dose of drugs with high mitochondrial toxicity or modifying the delivery system to allow controlled release may reduce the oxidative burden on the mother and fetus. A careful risk–benefit analysis is essential, particularly for drugs like ribavirin with teratogenic potential, where risks to fetal health often outweigh the benefits. In such cases, safer alternatives or stricter monitoring should be prioritized to protect fetal development. Long-term monitoring of infants exposed to antivirals in utero is also important. Antioxidants alongside antiviral therapy show promise in reducing oxidative stress, offering a potential strategy to balance effective antiviral use with fetal protection. Understanding oxidative stress’s role in neonatal outcomes is key to developing safer therapies, and future treatments should focus on reducing mitochondrial dysfunction and ensuring thorough safety evaluations in pregnant populations.

**Table 3:** Effects of antiviral drugs on oxidative stress.

Mechanism	Antiviral Class/Example	Effects in Pregnancy	Implications for Newborns
Directly Generate ROS	Zidovudine (NRTI), efavirenz (NNRTI), protease inhibitors	Mitochondrial dysfunction, increased oxidative stress, potential fetal growth restriction	Increased ROS production, potential for low birth weight, and neurodevelopmental risks
Impair Antioxidant Defenses	Integrase inhibitors (dolutegravir, raltegravir), neuraminidase inhibitors (oseltamivir)	Disruption of redox balance, compromised placental antioxidant activity	Reduced antioxidant capacity, higher risk of oxidative stress-related conditions

## 5. Efficacy and Safety of Specific Antioxidants in Reducing Oxidative Stress During Pregnancy

Several antioxidants have demonstrated potential in reducing oxidative stress-related complications during pregnancy, particularly in individuals undergoing antiviral treatments [56,141]. N-acetylcysteine (NAC) is a potent antioxidant that replenishes intracellular glutathione levels. NAC has been widely studied for its protective effects against drug-induced oxidative stress and has shown promise in preventing preterm birth, improving birth weight, and addressing recurrent pregnancy loss [142,143]. In addition to NAC, supplementation with vitamins C and E—well-known antioxidants that scavenge free radicals—has been suggested to reduce oxidative stress markers. This, in turn, may help prevent complications such as preeclampsia and IUGR, although results across studies remain inconsistent [144,145]. The findings from large-scale randomized controlled trials, such as vitamin C and vitamin E in pregnant women at risk for pre-eclampsia (VIP trial), indicate that antioxidant supplementation does not significantly reduce the incidence of preeclampsia or other adverse pregnancy outcomes [146]. For instance, the VIP trial showed that women receiving vitamin C (1000 mg) and vitamin E (400 IU) did not experience a lower rate of preeclampsia compared to those receiving a placebo. In fact, the intervention group exhibited a higher risk of gestational hypertension and adverse neonatal outcomes, such as low birth weight [147,148]. Moreover, systematic reviews and meta-analyses have consistently reported no significant benefits of antioxidant supplementation in preventing preeclampsia, severe preeclampsia, preterm birth, or neonatal death when compared to placebo groups. While meta-analyses indicate that vitamin C supplementation does not significantly reduce the incidence of preeclampsia or other adverse pregnancy outcomes, recent research has explored its role as an adjunctive treatment in managing oxidative stress during COVID-19. Studies have suggested that high-dose vitamin C may help modulate immune responses and reduce inflammation in non-pregnant populations. However, it is important to note that these findings have not yet been studied in pregnant individuals or newborns. This gap underscores the need for further investigation into the safety and efficacy of vitamin C therapy in these vulnerable populations.

This suggests that the anticipated protective effects of antioxidants against oxidative stress during pregnancy may not translate into clinical benefits. Regarding safety, while antioxidants are generally considered safe and have a low incidence of severe adverse effects, the results from clinical trials raise concerns about their use in specific populations. The increased risk of gestational hypertension and adverse neonatal outcomes observed in some studies indicate that antioxidant supplementation may not be without risks. Fabrizio et al. emphasize that despite the theoretical safety of vitamins, the clinical implications of their supplementation during pregnancy warrant caution [149]. The lack of demonstrated efficacy combined with potential risks suggests that healthcare providers should carefully consider the use of antioxidant supplements in pregnant women, particularly those at risk for complications like preeclampsia [7]. Fabrizio et al. also suggest that research should focus on the timing and context of supplementation rather than continuing to pursue trials that have not yielded positive results in the past. Another antioxidant, resveratrol, has been recognized for its potential to improve placental function and fetal growth and mitigate complications associated with gestational diabetes and maternal obesity [150,151]. However, evidence from human studies remains limited and contradictory [152]. Resveratrol intake has been shown to decrease inflammation and oxidative stress in placental and embryonic tissues, which are critical factors in adverse pregnancy outcomes [152,153]. Low doses may provide beneficial effects, while higher doses could potentially lead to adverse outcomes, suggesting the need for careful dosage considerations in supplementation.

Melatonin has shown promising effects in addressing placental insufficiency and related complications. It enhances antioxidant capacity in the placenta by upregulating antioxidant enzymes like thioredoxin, glutamate–cysteine ligase, and manganese SOD [154,155]. Melatonin also reduces oxidative stress by inhibiting NADPH- and iron-dependent lipid peroxidation in placental mitochondria [156]. In undernourished pregnancies, melatonin improves placental efficiency and birth weight [155]. It may reduce soluble fms-like tyrosine kinase-1 secretion from trophoblasts, potentially benefiting preeclampsia management [154]. Pregnancies complicated by placental insufficiency show altered melatonin secretion patterns, with lower systemic and placental concentrations and reduced receptor expression [157]. While small intervention studies suggest melatonin treatment may prolong pregnancy and improve outcomes, large-scale randomized controlled trials are still needed to confirm its efficacy [157].

Similarly, curcumin, the main polyphenol in turmeric, has shown promising effects in animal studies for improving pregnancy outcomes, particularly in complications like IUGR [158]. Its anti-inflammatory, antioxidant, and antiangiogenic properties present a potential therapeutic agent for various pregnancy-related disorders, including gestational diabetes mellitus, preeclampsia, and fetal growth disorders [159]. Curcumin’s pleiotropic functions and safety profile have led to increased interest in its use during pregnancy [160]. Curcumin has demonstrated beneficial effects on various chronic diseases in humans [161]. Nevertheless, research on its impact during human pregnancy remains limited. Current evidence originates from animal models and in vitro studies, highlighting the need for further investigation in human clinical trials to fully understand curcumin’s potential benefits and risks in pregnancy [159,160].

**Figure 2:**
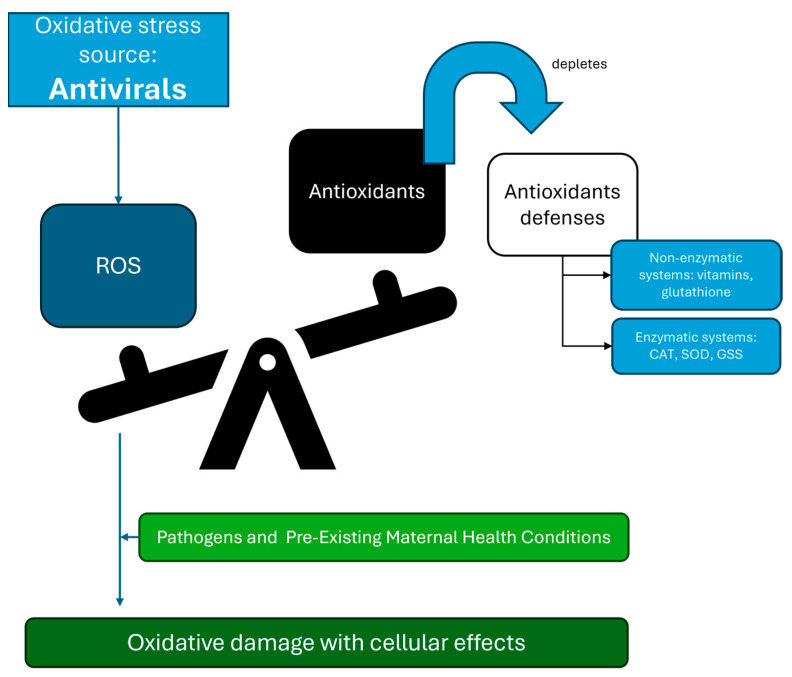
Illustration of the interplay between oxidative stress and antioxidant defenses.

Understanding oxidative stress mechanisms during pregnancy and their interactions with antiviral therapies is crucial for developing safer and more effective treatments for pregnant patients. Antiviral drugs can influence placental antioxidant systems by depleting essential antioxidants or disrupting pathways that mitigate oxidative stress (Figure 2). Therefore, selecting antivirals that support placental health and minimize oxidative damage is critical. Several antiviral drugs may have inherent antioxidant properties, providing an opportunity to create synergistic combinations that treat viral infections and reduce oxidative stress. This approach can develop multi-functional therapies that are effective and safe for the mother and fetus.

Minimizing the impact of antivirals on fetal oxidative stress is equally important for reducing developmental complications. Certain drugs may cross the placental barrier and affect the fetus’s antioxidant defenses. Selecting antivirals with minimal effects on fetal oxidative stress can lower the risk of developmental issues. Implementing targeted drug selection, developing combination therapies including antioxidants, and regularly monitoring oxidative stress markers during antiviral treatment are key strategies to prevent adverse pregnancy outcomes.

To optimize antiviral regimens for pregnant patients, comprehensive research is needed to evaluate the effects of various antivirals on placental and fetal oxidative stress mechanisms. Developing antioxidant-enhanced antivirals and exploring combination therapies with antioxidants can help mitigate oxidative stress-related risks. Additionally, identifying biomarkers of oxidative stress will guide treatment decisions and therapy effectiveness. Finally, dosing strategies should be optimized to balance antiviral efficacy with minimal oxidative stress, ensuring safer treatments for pregnant patients and their developing infants.

## 6. Potential Strategies to Mitigate Antiviral-Induced Oxidative Stress

Here, several proposed strategies to advance research on assessing oxidative stress induced by antiviral drugs are discussed. In vulnerable populations, such as pregnant patients, both the viral infection and pregnancy itself can increase oxidative stress. Therefore, antiviral drugs should be carefully selected to avoid adding further oxidative burden. The goal is to ensure that treatment does not exacerbate oxidative stress while effectively managing the viral infection.

Pharmacological agents targeting mitochondrial antioxidant systems and inducing antioxidant enzyme expression could be a promising strategy to mitigate antiviral-induced oxidative stress during pregnancy. Mitochondria-targeted antioxidants, particularly MitoQ and SkQ1, have shown promise in addressing oxidative stress-related conditions, including pregnancy complications [53]. These compounds accumulate in mitochondria, the primary cellular ROS source, providing targeted protection against oxidative damage [162]. They have demonstrated beneficial effects in various animal models and some clinical trials [53]. However, timing of administration is crucial, as MitoQ protects against preeclampsia when given in late gestation but exacerbates the condition when administered early in pregnancy. This occurs due to mild oxidative stress that is necessary for proper placentation [163,164]. Mitochondria-targeted antioxidants act by preventing chain reactions of cardiolipin peroxidation initiated by mitochondrial ROS, which play a key role in many degenerative processes. These types of antioxidants could help preserve placental function and fetal development. Stimulating the expression of antioxidant enzymes like SOD and CAT can enhance the body’s natural defense mechanisms against oxidative stress preparing cells to better cope with increased oxidative stress from antiviral therapy, providing sustained protection against various forms of oxidative damage. Combining enzyme inducers with mitochondria-targeted antioxidants could offer comprehensive protection against oxidative stress [165]. Adjunctive therapies like coenzyme Q10 (CoQ10) and alpha-lipoic acid also provide potent antioxidant support, reducing oxidative stress by regenerating other antioxidants and protecting mitochondrial function [166]. Combining these strategies can help mitigate oxidative stress induced by antiviral therapies, thus improving patient outcomes and quality of life. Mechanistic insights into how specific antivirals induce oxidative stress at the cellular level are critical. Such studies can identify which drugs or drug combinations are most likely to increase ROS production, allowing for the design of safer therapeutic regimens or adjunctive antioxidant therapies. Optimizing dosing strategies based on insights into oxidative stress mechanisms is another crucial approach, as it allows for balancing antiviral efficacy while minimizing oxidative harm.

The approach that involves targeting cellular oxidoreductases and the modulation of the Nrf2 pathway might also be beneficial. Cellular oxidoreductases play a crucial role in viral entry and infection-associated oxidative stress, rendering them potential targets for antiviral strategies [167]. Viruses can modulate the Nrf2 pathway, affecting viral replication and disease progression [168]. Studies have shown an inverse relationship between Nrf2 expression and viral entry/replication, with Nrf2 knockdown increasing influenza virus susceptibility and Nrf2 activation via compounds like sulforaphane and epigallocatechin gallate decreasing viral entry and replication [169]. These findings suggest that targeting oxidoreductases with inhibitors and modulating the Nrf2 pathway could be promising approaches for suppressing viral replication and oxidative damage [167,170]. However, careful assessment of benefits and risks is necessary when considering these antiviral strategies [167]. Regular assessment of oxidative stress markers and antioxidant levels can help identify early signs of oxidative damage, enabling timely interventions to minimize complications. The development of oxidative stress biomarkers is key to personalized medicine in pregnant patients receiving antiviral therapy. Real-time monitoring of oxidative stress levels can guide dose adjustments and reduce the risk of oxidative damage. Additionally, combining antivirals with antioxidant supplements can further reduce the oxidative burden, ensuring safer antiviral regimens [171]. Ongoing clinical trials that include oxidative stress measurements, as well as longitudinal studies tracking children exposed to these drugs in utero, can provide valuable insights into the long-term impact of oxidative stress, guiding future treatment protocols.

Advancements in the study of viral infections, preeclampsia, and IUGR have identified oxidative stress as a shared underlying mechanism driving these conditions. Recent approaches to treating preeclampsia and IUGR have focused on addressing oxidative stress. In the context of viral infections, these may help mitigate the oxidative damage caused by both viral infections and antiviral treatments during pregnancy. Developing antivirals that minimize oxidative stress could potentially reduce the risk of preeclampsia and IUGR in infected pregnant individuals [172].

Nutrigenetic studies are exploring how genetic variations affect an individual’s response to nutrients and their susceptibility to oxidative stress. In the context of viral infections and pregnancy complications, we can have: (1) Personalized nutrition: tailoring dietary interventions based on genetic profiles could help pregnant individuals better manage oxidative stress induced by viral infections. (2) Antioxidant supplementation: identifying genetic markers that predict responsiveness to antioxidant supplementation could help prevent preeclampsia and IUGR in virus-infected pregnant individuals [173].

Recent research into new preventive drugs for preeclampsia and IUGR highlights potential connections to viral infections. Therapeutics that activate the Nrf2 pathway, a key regulator of the antioxidative response, hold promise for counteracting virus-induced oxidative stress and reducing the risk of pregnancy complications. Additionally, mitochondrial-targeted therapies may offer another avenue for intervention, as viruses such as SARS-CoV-2 are known to disrupt mitochondrial function. By preserving mitochondrial health, these therapies could help mitigate oxidative stress-related complications during pregnancy [174].

Future research should focus on investigating the long-term effects of maternal viral infections on placental function and fetal development, developing antiviral treatments that minimize oxidative stress during pregnancy, and exploring the potential of personalized medicine approaches in managing viral infections and preventing related pregnancy complications. Moreover, in vitro studies on oxidative stress and antiviral medications can provide valuable insights that inform the development and refinement of DILIsym X (DSX) software. DILIsym is a computational tool used to predict drug-induced liver injury (DILI) and other adverse drug reactions. The use of DILIsym software can further benefit research by integrating in vitro data on oxidative stress and antiviral medications [175]. In vitro studies provide mechanistic insights into oxidative stress pathways, helping researchers understand how antiviral drugs interact with cellular components and antioxidant defenses [75]. In DILIsym, several in vitro input values can help simulate the interactions and effects of drugs on liver function. Some key in vitro measurements that can be incorporated into DILIsym include Mitochondrial Function (mitochondrial membrane potential, ATP levels, ROS production, mitochondrial mass), Cell Health Indicators (cell viability, apoptosis and necrosis markers, cellular ATP content), Oxidative Stress Markers (GSH levels, ROS levels, Lipid peroxidation), Enzyme and Transporter Activity (bile acid transporter inhibition (e.g., BSEP, MRP3/4), enzyme inhibition (e.g., UGT1A1)), Biomarkers (traditional liver injury biomarkers (e.g., ALT, AST), novel biomarkers (e.g., HMGB1, K18)), Metabolic Parameters (metabolic rate of the drug, formation of reactive metabolites). These in vitro measurements help DILIsym simulate the dose–response relationship and predict potential hepatotoxicity in vivo [176]. This information is vital for simulating drug effects in DILIsym, especially in defining dose–response relationships and identifying threshold levels at which oxidative stress becomes significant. In vitro experiments are crucial to identifying reliable oxidative stress biomarkers, which can be integrated into DILIsym to enhance predictive accuracy and ensure safer drug regimens. These experiments help determine the dose–response relationship between antiviral drug concentrations and oxidative stress markers, essential for accurate DILIsym simulation. Identifying threshold levels of oxidative stress allows for the establishment of safety margins within the software [177].

Integrating physiologically based pharmacokinetic (PBPK) models, such as those in GastroPlus and PK-Sim, is crucial for simulating drug exposure during pregnancy [178]. These models predict antiviral drug interactions within the complex physiological environment of pregnancy, aiding in the optimization of dosing strategies to balance viral suppression and minimize oxidative stress-related complications. In vitro research also maps the metabolic pathways of antiviral drugs, including the generation of reactive metabolites that contribute to oxidative stress [179]. Studying drug interactions at the cellular level can predict potential adverse effects from concurrent medication use. Additionally, in silico drug combination assays using GastroPlus or PK-Sim can simulate drug–drug interactions, further optimizing therapy [180,181]. Researchers can use in vitro findings to test various scenarios in DILIsym, such as different dosing regimens or patient populations, to predict outcomes more accurately.

## 7. Conclusions

The literature indicates that antiretrovirals, particularly zidovudine, can cause mitochondrial dysfunction, a major source of oxidative stress. This dysfunction may impair energy production and lead to the accumulation of ROS. In the placenta, oxidative stress can compromise function and lead to complications such as preeclampsia, IUGR, and other adverse outcomes. Oxidative stress may also impact fetal brain development, with animal studies showing impaired cognitive functions following prenatal exposure to antiretrovirals. Furthermore, oxidative stress is linked to conditions like small gestational age and low birth weight, critical indicators of fetal health. Increased oxidative stress has also been associated with preterm labor, contributing to complications like respiratory distress and long-term developmental issues in newborns. Antiretrovirals are crucial for managing HIV during pregnancy, but ARV also poses risks related to oxidative stress. Studies on the oxidative effects of antiretrovirals provide insight into how specific drugs and dosages lead to increased ROS production and mitochondrial dysfunction. Understanding these mechanisms is essential for identifying which drugs or combinations are most likely to induce oxidative stress. By pinpointing the pathways through which antiretrovirals cause oxidative stress, researchers can develop therapies that either avoid these pathways or incorporate adjunctive treatments, such as antioxidants, to counteract these effects. Insights gained from oxidative stress studies can be integrated into clinical guidelines, helping healthcare providers select the safest and most effective ART regimens for pregnant women. This could include specific recommendations on drug choices, dosage adjustments, and monitoring protocols. These guidelines can also include risk management strategies, such as more frequent monitoring of oxidative stress markers in women on high-risk regimens or adjusting therapy based on oxidative stress levels.

## Figures and Tables

**Table 1:** Challenges in assessing oxidative stress related to antiviral treatments.

Challenges in Assessing Oxidative Stress with Antivirals		Details
Complexity of Oxidative Stress Mechanisms	Multiple ROS Sources	Antivirals can induce ROS production through different mechanisms (e.g., mitochondrial dysfunction, ER stress), making it challenging to pinpoint the exact source.
	Antioxidant System Variability	Individual differences in antioxidant defense systems can affect the overall oxidative stress response to antivirals.
Temporal Dynamics	Acute vs. Chronic Effects	Short-term oxidative stress may differ significantly from long-term effects, requiring longitudinal studies to capture the full impact of antiviral therapy.
	Adaptive Responses	The body may adapt to oxidative stress over time, potentially masking the true extent of damage in long-term assessments.
Measurement Challenges (Biomarker limitations)	Specificity	Many oxidative stress biomarkers lack specificity to particular ROS or antioxidants, making it difficult to attribute changes to specific antiviral drugs.
	Stability	Some oxidative stress markers are unstable and can be affected by sample handling and storage conditions.
	Methodological Issues	Invasiveness: Direct measurement of ROS often requires invasive procedures, limiting their applicability in clinical settings.
	Indirect Measurements	Many assessments rely on indirect markers of oxidative damage (e.g., lipid peroxidation products), which may not always accurately reflect the current oxidative state.
Confounding Factors	Underlying Disease State	The viral infection itself can induce oxidative stress, making it challenging to distinguish drug-induced effects from disease-related oxidative stress.
	Lifestyle Factors	Diet, exercise, and other lifestyle factors can significantly influence oxidative stress levels, potentially confounding the assessment of antiviral-induced effects.
Variability in Drug Responses	Pharmacogenomics	Genetic variations can affect how individuals metabolize antivirals, leading to differences in drug-induced oxidative stress.
	Drug Interactions	Many patients receive multiple medications, which can interact and affect oxidative stress levels in unpredictable ways.
Tissue-Specific Effects	Localized vs. Systemic Effects	Antivirals may induce oxidative stress in specific tissues or organs, which may not be reflected in systemic measurements
	Accessibility	Some tissues affected by oxidative stress may not be easily accessible for direct measurement.
Technological Limitations	In vivo vs. In vitro Discrepancies	Results from cell culture studies may not accurately reflect the complex in vivo environment.
	Real-Time Monitoring	Current technologies often lack the ability to provide real-time, continuous monitoring of oxidative stress in clinical settings.

## References

[1] Money D.M. (2003). Antiviral and Antiretroviral Use in Pregnancy. Obstet. Gynecol. Clin. N. Am..

[2] Waitt C., Astill D., Zavala E., Karron R.A., Faden R.R., Stratton P., Temkin S.M., Clayton J.A. (2022). Clinical Trials and Pregnancy. Commun. Med..

[3] Sewell C.A., Sheehan S.M., Gill M.S., Henry L.M., Bucci-Rechtweg C., Gyamfi-Bannerman C., Lyerly A.D., McKinney L.C., Hatfield K.P., Baer G.R. (2022). Scientific, Ethical, and Legal Considerations for the Inclusion of Pregnant People in Clinical Trials. Am. J. Obstet. Gynecol..

[4] Recommendations for the Use of Antiretroviral Drugs During Pregnancy. https://clinicalinfo.hiv.gov/en/guidelines/perinatal/recommendations-arv-drugs-pregnancy-overview#:~:text=Overview,-Panel’s%20Recommendations&text=All%20pregnant%20people%20with%20HIV,and%20sexual%20transmission%20(AI).

[5] Kolding L., Eken H., Uldbjerg N. (2020). Drug Exposure during Pregnancy and Fetal Cardiac Function—A Systematic Review. J. Perinat. Med..

[6] Bérard A., Sheehy O., Zhao J., Vinet É., Bernatsky S., Abrahamowicz M. (2017). SSRI and SNRI Use during Pregnancy and the Risk of Persistent Pulmonary Hypertension of the Newborn. Br. J. Clin. Pharmacol..

[7] Odufalu F.-D., Long M., Lin K., Mahadevan U. (2022). Exposure to Corticosteroids in Pregnancy Is Associated with Adverse Perinatal Outcomes among Infants of Mothers with Inflammatory Bowel Disease: Results from the PIANO Registry. Gut.

[8] Ross E.J., Graham D.L., Money K.M., Stanwood G.D. (2015). Developmental Consequences of Fetal Exposure to Drugs: What We Know and What We Still Must Learn. Neuropsychopharmacology.

[9] Hudson R.E., Metz T.D., Ward R.M., McKnite A.M., Enioutina E.Y., Sherwin C.M., Watt K.M., Job K.M. (2023). Drug Exposure during Pregnancy: Current Understanding and Approaches to Measure Maternal-Fetal Drug Exposure. Front. Pharmacol..

[10] Ibrahim A., Khoo M.I., Ismail E.H.E., Hussain N.H.N., Zin A.A.M., Noordin L., Abdullah S., Mahdy Z.A., Lah N.A.Z.N. (2024). Oxidative Stress Biomarkers in Pregnancy: A Systematic Review. Reprod. Biol. Endocrinol..

[11] Pereira A.C., Martel F. (2014). Oxidative Stress in Pregnancy and Fertility Pathologies. Cell Biol. Toxicol..

[12] Zhang C., Guo Y., Yang Y., Du Z., Fan Y., Zhao Y., Yuan S. (2023). Oxidative Stress on Vessels at the Maternal-Fetal Interface for Female Reproductive System Disorders: Update. Front. Endocrinol..

[13] Moreno-Fernandez J., Ochoa J.J., De Paco Matallana C., Caño A., Martín-Alvarez E., Sanchez-Romero J., Toledano J.M., Puche-Juarez M., Prados S., Ruiz-Duran S. (2022). COVID-19 during Gestation: Maternal Implications of Evoked Oxidative Stress and Iron Metabolism Impairment. Antioxidants.

[14] Mandò C., Savasi V.M., Anelli G.M., Corti S., Serati A., Lisso F., Tasca C., Novielli C., Cetin I. (2021). Mitochondrial and Oxidative Unbalance in Placentas from Mothers with SARS-CoV-2 Infection. Antioxidants.

[15] Ighodaro O.M., Akinloye O.A. (2018). First Line Defence Antioxidants-Superoxide Dismutase (SOD), Catalase (CAT) and Glutathione Peroxidase (GPX): Their Fundamental Role in the Entire Antioxidant Defence Grid. Alex. J. Med..

[16] Vornic I., Buciu V., Furau C.G., Gaje P.N., Ceausu R.A., Dumitru C.-S., Barb A.C., Novacescu D., Cumpanas A.A., Latcu S.C. (2024). Oxidative Stress and Placental Pathogenesis: A Contemporary Overview of Potential Biomarkers and Emerging Therapeutics. Int. J. Mol. Sci..

[17] Garcia-Flores V., Romero R., Xu Y., Theis K.R., Arenas-Hernandez M., Miller D., Peyvandipour A., Bhatti G., Galaz J., Gershater M. (2022). Maternal-Fetal Immune Responses in Pregnant Women Infected with SARS-CoV-2. Nat. Commun..

[18] Argueta L.B., Lacko L.A., Bram Y., Tada T., Carrau L., Rendeiro A.F., Zhang T., Uhl S., Lubor B.C., Chandar V. (2022). Inflammatory Responses in the Placenta upon SARS-CoV-2 Infection Late in Pregnancy. iScience.

[19] Hussain T., Murtaza G., Metwally E., Kalhoro D.H., Kalhoro M.S., Rahu B.A., Sahito R.G.A., Yin Y., Yang H., Chughtai M.I. (2021). The Role of Oxidative Stress and Antioxidant Balance in Pregnancy. Mediat. Inflamm..

[20] Nüsken E., Appel S., Saschin L., Kuiper-Makris C., Oberholz L., Schömig C., Tauscher A., Dötsch J., Kribs A., Alejandre Alcazar M.A. (2024). Intrauterine Growth Restriction: Need to Improve Diagnostic Accuracy and Evidence for a Key Role of Oxidative Stress in Neonatal and Long-Term Sequelae. Cells.

[21] Lee C. (2018). Therapeutic Modulation of Virus-Induced Oxidative Stress via the Nrf2-Dependent Antioxidative Pathway. Oxid. Med. Cell. Longev..

[22] Gain C., Song S., Angtuaco T., Satta S., Kelesidis T. (2023). The Role of Oxidative Stress in the Pathogenesis of Infections with Coronaviruses. Front. Microbiol..

[23] Silwal P., Kim J.K., Kim Y.J., Jo E.-K. (2020). Mitochondrial Reactive Oxygen Species: Double-Edged Weapon in Host Defense and Pathological Inflammation During Infection. Front. Immunol..

[24] Prasada Kabekkodu S., Chakrabarty S., Jayaram P., Mallya S., Thangaraj K., Singh K.K., Satyamoorthy K. (2023). Severe Acute Respiratory Syndrome Coronaviruses Contributing to Mitochondrial Dysfunction: Implications for Post-COVID Complications. Mitochondrion.

[25] Schank M., Zhao J., Wang L., Nguyen L.N.T., Cao D., Dang X., Khanal S., Zhang J., Zhang Y., Wu X.Y. (2021). Oxidative Stress Induces Mitochondrial Compromise in CD4 T Cells From Chronically HCV-Infected Individuals. Front. Immunol..

[26] Shimizu I., Shimamoto N., Saiki K., Furujo M., Osaw K. (2012). Lipid Peroxidation in Hepatic Fibrosis. Lipid Peroxidation.

[27] Zhou Y., Long D., Zhao Y., Li S., Liang Y., Wan L., Zhang J., Xue F., Feng L. (2022). Oxidative Stress-Mediated Mitochondrial Fission Promotes Hepatic Stellate Cell Activation via Stimulating Oxidative Phosphorylation. Cell Death Dis..

[28] Rani R., Gandhi C.R. (2023). Stellate Cell in Hepatic Inflammation and Acute Injury. J. Cell Physiol..

[29] Baghaei K., Mazhari S., Tokhanbigli S., Parsamanesh G., Alavifard H., Schaafsma D., Ghavami S. (2022). Therapeutic Potential of Targeting Regulatory Mechanisms of Hepatic Stellate Cell Activation in Liver Fibrosis. Drug Discov. Today.

[30] Ivanov A., Bartosch B., Smirnova O., Isaguliants M., Kochetkov S. (2013). HCV and Oxidative Stress in the Liver. Viruses.

[31] Liu M., Chen F., Liu T., Chen F., Liu S., Yang J. (2017). The Role of Oxidative Stress in Influenza Virus Infection. Microbes Infect..

[32] Kim C.-U., Lim D., Kim Y.S., Ku B., Kim D.-J. (2023). Influenza Viral Matrix 1 Protein Aggravates Viral Pathogenicity by Inducing TLR4-Mediated Reactive Oxygen Species Production and Apoptotic Cell Death. Cell Death Dis..

[33] Ivanov A.V., Valuev-Elliston V.T., Ivanova O.N., Kochetkov S.N., Starodubova E.S., Bartosch B., Isaguliants M.G. (2016). Oxidative Stress during HIV Infection: Mechanisms and Consequences. Oxid. Med. Cell. Longev..

[34] Buckley S., Byrnes S., Cochrane C., Roche M., Estes J.D., Selemidis S., Angelovich T.A., Churchill M.J. (2021). The Role of Oxidative Stress in HIV-Associated Neurocognitive Disorders. Brain Behav. Immun. Health.

[35] Ngo V., Duennwald M.L. (2022). Nrf2 and Oxidative Stress: A General Overview of Mechanisms and Implications in Human Disease. Antioxidants.

[36] Hammad M., Raftari M., Cesário R., Salma R., Godoy P., Emami S.N., Haghdoost S. (2023). Roles of Oxidative Stress and Nrf2 Signaling in Pathogenic and Non-Pathogenic Cells: A Possible General Mechanism of Resistance to Therapy. Antioxidants.

[37] Cuadrado A., Pajares M., Benito C., Jiménez-Villegas J., Escoll M., Fernández-Ginés R., Garcia Yagüe A.J., Lastra D., Manda G., Rojo A.I. (2020). Can Activation of NRF2 Be a Strategy against COVID-19?. Trends Pharmacol. Sci..

[38] Kombe Kombe A.J., Fotoohabadi L., Nanduri R., Gerasimova Y., Daskou M., Gain C., Sharma E., Wong M., Kelesidis T. (2024). The Role of the Nrf2 Pathway in Airway Tissue Damage Due to Viral Respiratory Infections. Int. J. Mol. Sci..

[39] Kalantari L., Ghotbabadi Z.R., Gholipour A., Ehymayed H.M., Najafiyan B., Amirlou P., Yasamineh S., Gholizadeh O., Emtiazi N. (2023). A State-of-the-Art Review on the NRF2 in Hepatitis Virus-Associated Liver Cancer. Cell Commun. Signal..

[40] Waqas F.H., Shehata M., Elgaher W.A.M., Lacour A., Kurmasheva N., Begnini F., Kiib A.E., Dahlmann J., Chen C., Pavlou A. (2023). NRF2 Activators Inhibit Influenza A Virus Replication by Interfering with Nucleo-Cytoplasmic Export of Viral RNPs in an NRF2-Independent Manner. PLoS Pathog..

[41] Hamad R.S., Al-kuraishy H.M., Alexiou A., Papadakis M., Ahmed E.A., Saad H.M., Batiha G.E.-S. (2023). SARS-CoV-2 Infection and Dysregulation of Nuclear Factor Erythroid-2-Related Factor 2 (Nrf2) Pathway. Cell Stress. Chaperones.

[42] Ramezani A., Nahad M.P., Faghihloo E. (2018). The Role of Nrf2 Transcription Factor in Viral Infection. J. Cell Biochem..

[43] Herengt A., Thyrsted J., Holm C.K. (2021). NRF2 in Viral Infection. Antioxidants.

[44] Cornish E.F., Filipovic I., Åsenius F., Williams D.J., McDonnell T. (2020). Innate Immune Responses to Acute Viral Infection During Pregnancy. Front. Immunol..

[45] Megli C.J., Coyne C.B. (2022). Infections at the Maternal–Fetal Interface: An Overview of Pathogenesis and Defence. Nat. Rev. Microbiol..

[46] Cruz-Holguín V.J., González-García L.D., Velázquez-Cervantes M.A., Arévalo-Romero H., De Jesús-González L.A., Helguera-Repetto A.C., León-Reyes G., Salazar M.I., Cedillo-Barrón L., León-Juárez M. (2024). Collateral Damage in the Placenta during Viral Infection in Pregnancy: A Possible Mechanism for Vertical Transmission and an Adverse Pregnancy Outcome. Diseases.

[47] Beltrami S., Rizzo S., Schiuma G., Speltri G., Di Luca D., Rizzo R., Bortolotti D. (2023). Gestational Viral Infections: Role of Host Immune System. Microorganisms.

[48] Espino A., El Costa H., Tabiasco J., Al-Daccak R., Jabrane-Ferrat N. (2021). Innate Immune Response to Viral Infections at the Maternal-Fetal Interface in Human Pregnancy. Front. Med..

[49] Tossetta G., Fantone S., Piani F., Crescimanno C., Ciavattini A., Giannubilo S.R., Marzioni D. (2023). Modulation of NRF2/KEAP1 Signaling in Preeclampsia. Cells.

[50] Fields N.J., Palmer K.R., Nisi A., Marshall S.A. (2023). Preeclampsia to COVID-19: A Journey towards Improved Placental and Vascular Function Using Sulforaphane. Placenta.

[51] Loboda A., Damulewicz M., Pyza E., Jozkowicz A., Dulak J. (2016). Role of Nrf2/HO-1 System in Development, Oxidative Stress Response and Diseases: An Evolutionarily Conserved Mechanism. Cell. Mol. Life Sci..

[52] Khan M.Z., Khan A., Huang B., Wei R., Kou X., Wang X., Chen W., Li L., Zahoor M., Wang C. (2024). Bioactive Compounds Protect Mammalian Reproductive Cells from Xenobiotics and Heat Stress-Induced Oxidative Distress via Nrf2 Signaling Activation: A Narrative Review. Antioxidants.

[53] Zinovkin R.A., Zamyatnin A.A. (2019). Mitochondria-Targeted Drugs. Curr. Mol. Pharmacol..

[54] Stoker M.L., Newport E., Hulit J.C., West A.P., Morten K.J. (2019). Impact of Pharmacological Agents on Mitochondrial Function: A Growing Opportunity?. Biochem. Soc. Trans..

[55] Zhang Q., Meng Y., Wang K., Zhang X., Chen W., Sheng J., Qiu Y., Diao H., Li L. (2021). Inflammation and Antiviral Immune Response Associated With Severe Progression of COVID-19. Front. Immunol..

[56] Sebastiani G., Navarro-Tapia E., Almeida-Toledano L., Serra-Delgado M., Paltrinieri A.L., García-Algar Ó., Andreu-Fernández V. (2022). Effects of Antioxidant Intake on Fetal Development and Maternal/Neonatal Health during Pregnancy. Antioxidants.

[57] Chow E.J., Beigi R.H., Riley L.E., Uyeki T.M. (2021). Clinical Effectiveness and Safety of Antivirals for Influenza in Pregnancy. Open Forum Infect. Dis..

[58] Pan X., Chen J., Zhou L., Ou X., He F., Liu Y., Zheng S., Wang H., Cao B., Wang Z. (2020). Efficacy and Safety of Continuous Antiviral Therapy from Preconception to Prevent Perinatal Transmission of Hepatitis B Virus. Sci. Rep..

[59] Wang L.-Y., Cui J.-J., OuYang Q.-Y., Zhan Y., Wang Y.-M., Xu X.-Y., Yu L.-L., Yin H., Wang Y., Luo C.-H. (2021). Complex Analysis of the Personalized Pharmacotherapy in the Management of COVID-19 Patients and Suggestions for Applications of Predictive, Preventive, and Personalized Medicine Attitude. EPMA J..

[60] Dopazo J., Maya-Miles D., García F., Lorusso N., Calleja M.Á., Pareja M.J., López-Miranda J., Rodríguez-Baño J., Padillo J., Túnez I. (2021). Implementing Personalized Medicine in COVID-19 in Andalusia: An Opportunity to Transform the Healthcare System. J. Pers. Med..

[61] Akbari H., Taghizadeh-Hesary F. (2023). COVID-19 Induced Liver Injury from a New Perspective: Mitochondria. Mitochondrion.

[62] FakhriRavari A., Malakouti M. (2024). Remdesivir and the Liver: A Concise Narrative Review of Remdesivir-Associated Hepatotoxicity in Patients Hospitalized Due to COVID-19. Pharmacoepidemiology.

[63] Aleem A., Mahadevaiah G., Shariff N., Kothadia J.P. (2021). Hepatic Manifestations of COVID-19 and Effect of Remdesivir on Liver Function in Patients with COVID-19 Illness. Bayl. Univ. Med. Cent. Proc..

[64] Abraham P., Ramamoorthy H., Isaac B. (2013). Depletion of the Cellular Antioxidant System Contributes to Tenofovir Disoproxil Fumarate—Induced Mitochondrial Damage and Increased Oxido-Nitrosative Stress in the Kidney. J. Biomed. Sci..

[65] Ramamoorthy H., Abraham P., Isaac B., Selvakumar D. (2017). Role for NF-ΚB Inflammatory Signalling Pathway in Tenofovir Disoproxil Fumarate (TDF) Induced Renal Damage in Rats. Food Chem. Toxicol..

[66] Cheng P.-N., Sun H.-Y., Feng I.-C., Wang S.-T., Chiu Y.-C., Chiu H.-C., Chien S.-C., Young K.-C. (2023). Reversibility of Some Oxidative Stress Markers in Chronic Hepatitis C Patients after Receiving Direct-Acting Antiviral Agents. J. Virus Erad..

[67] Reyskens K.M.S.E., Essop M.F. (2014). HIV Protease Inhibitors and Onset of Cardiovascular Diseases: A Central Role for Oxidative Stress and Dysregulation of the Ubiquitin–Proteasome System. Biochim. Et Biophys. Acta (BBA)—Mol. Basis Dis..

[68] Proskurnina E.V., Izmailov D.Y., Sozarukova M.M., Zhuravleva T.A., Leneva I.A., Poromov A.A. (2020). Antioxidant Potential of Antiviral Drug Umifenovir. Molecules.

[69] Camini F.C., da Silva T.F., da Silva Caetano C.C., Almeida L.T., Ferraz A.C., Alves Vitoreti V.M., de Mello Silva B., de Queiroz Silva S., de Magalhães J.C., de Brito Magalhães C.L. (2018). Antiviral Activity of Silymarin against Mayaro Virus and Protective Effect in Virus-Induced Oxidative Stress. Antiviral Res..

[70] Fedoreyev S.A., Krylova N.V., Mishchenko N.P., Vasileva E.A., Pislyagin E.A., Iunikhina O.V., Lavrov V.F., Svitich O.A., Ebralidze L.K., Leonova G.N. (2018). Antiviral and Antioxidant Properties of Echinochrome A. Mar. Drugs.

[71] Zhang Y., Wang Z., Chen H., Chen Z., Tian Y. (2014). Antioxidants: Potential Antiviral Agents for Japanese Encephalitis Virus Infection. Int. J. Infect. Dis..

[72] Vasil’ev A.N., Deriabin P.G., Galegov G.A. (2011). Antiviral Activity of Recombinant Interferon-Alpha-2b in Combination with Certain Antioxidant. Antibiot. Khimioter.

[73] Sutter J., Brettschneider J., Wigdahl B., Bruggeman P.J., Krebs F.C., Miller V. (2024). Non-Thermal Plasma Reduces HSV-1 Infection of and Replication in HaCaT Keratinocytes In Vitro. Int. J. Mol. Sci..

[74] Maldonado E., Morales-Pison S., Urbina F., Solari A. (2023). Aging Hallmarks and the Role of Oxidative Stress. Antioxidants.

[75] Jena A.B., Samal R.R., Bhol N.K., Duttaroy A.K. (2023). Cellular Red-Ox System in Health and Disease: The Latest Update. Biomed. Pharmacother..

[76] Jomova K., Raptova R., Alomar S.Y., Alwasel S.H., Nepovimova E., Kuca K., Valko M. (2023). Reactive Oxygen Species, Toxicity, Oxidative Stress, and Antioxidants: Chronic Diseases and Aging. Arch. Toxicol..

[77] Deavall D.G., Martin E.A., Horner J.M., Roberts R. (2012). Drug-Induced Oxidative Stress and Toxicity. J. Toxicol..

[78] Grzeszczak K., Łanocha-Arendarczyk N., Malinowski W., Ziętek P., Kosik-Bogacka D. (2023). Oxidative Stress in Pregnancy. Biomolecules.

[79] Díaz-Castro J., Toledano J.M., Sanchez-Romero J., Aguilar A.C., Martín-Alvarez E., Puche-Juarez M., Moreno-Fernandez J., Pinar-Gonzalez M., Prados S., Carrillo M.P. (2024). COVID-19 and Pregnancy: A Dangerous Mix for Bone Turnover and Metabolism Biomarkers in Placenta and Colostrum. J. Clin. Med..

[80] Rolfo A., Cosma S., Nuzzo A.M., Salio C., Moretti L., Sassoè-Pognetto M., Carosso A.R., Borella F., Cutrin J.C., Benedetto C. (2022). Increased Placental Anti-Oxidant Response in Asymptomatic and Symptomatic COVID-19 Third-Trimester Pregnancies. Biomedicines.

[81] Zhang C.X.W., Candia A.A., Sferruzzi-Perri A.N. (2024). Placental Inflammation, Oxidative Stress, and Fetal Outcomes in Maternal Obesity. Trends Endocrinol. Metab..

[82] Raffaeli G., Manzoni F., Cortesi V., Cavallaro G., Mosca F., Ghirardello S. (2020). Iron Homeostasis Disruption and Oxidative Stress in Preterm Newborns. Nutrients.

[83] Oseghale O., Vlahos R., O’Leary J.J., Brooks R.D., Brooks D.A., Liong S., Selemidis S. (2022). Influenza Virus Infection during Pregnancy as a Trigger of Acute and Chronic Complications. Viruses.

[84] Kotsias F., Hoffmann E., Amigorena S., Savina A. (2013). Reactive Oxygen Species Production in the Phagosome: Impact on Antigen Presentation in Dendritic Cells. Antioxid. Redox Signal.

[85] To E.E., Erlich J.R., Liong F., Liong S., Luong R., Oseghale O., Miles M.A., Papagianis P.C., Quinn K.M., Bozinovski S. (2022). Therapeutic Targeting of Endosome and Mitochondrial Reactive Oxygen Species Protects Mice From Influenza Virus Morbidity. Front. Pharmacol..

[86] Martinez Manfio V., Tasca K.I., Garcia J.L., de Oliveira Góis J., Correa C.R., de Souza L.d.R. (2021). Redox Imbalance Is Related to HIV and Pregnancy. PLoS ONE.

[87] Riggs P.K., Anderson A.M., Tang B., Rubin L.H., Morgello S., Marra C.M., Gelman B.B., Clifford D.B., Franklin D., Heaton R.K. (2023). Elevated Plasma Protein Carbonyl Concentration Is Associated with More Abnormal White Matter in People with HIV. Viruses.

[88] Paramasivam S., Krishnaswamy S., Giles M.L. (2023). Unravelling the Mechanisms by Which Chronic Hepatitis B Infection Is Associated with an Increased Risk of Gestational Diabetes. Front. Glob. Womens Health.

[89] Pergam S.A., Wang C.C., Gardella C.M., Sandison T.G., Phipps W.T., Hawes S.E. (2008). Pregnancy Complications Associated with Hepatitis C: Data from a 2003-2005 Washington State Birth Cohort. Am. J. Obstet. Gynecol..

[90] Afraie M., Moradi G., Zamani K., Azami M., Moradi Y. (2023). The Effect of Hepatitis B Virus on the Risk of Pregnancy Outcomes: A Systematic Review and Meta-Analysis of Cohort Studies. Virol. J..

[91] Bai H. (2007). Relationship of Hepatitis B Virus Infection of Placental Barrier and Hepatitis B Virus Intra-Uterine Transmission Mechanism. World J. Gastroenterol..

[92] León-Juárez M., Martínez–Castillo M., González-García L.D., Helguera-Repetto A.C., Zaga-Clavellina V., García-Cordero J., Flores-Pliego A., Herrera-Salazar A., Vázquez-Martínez E.R., Reyes-Muñoz E. (2017). Cellular and Molecular Mechanisms of Viral Infection in the Human Placenta. Pathog. Dis..

[93] Rose P.C., Nel E.D., Cotton M.F., Pitcher R.D., Otwombe K., Browne S.H., Innes S. (2022). Prevalence and Risk Factors for Hepatic Steatosis in Children With Perinatal HIV on Early Antiretroviral Therapy Compared to HIV-Exposed Uninfected and HIV-Unexposed Children. Front. Pediatr..

[94] Koi H., Zhang J., Makrigiannakis A., Getsios S., MacCalman C.D., Strauss J.F., Parry S. (2002). Syncytiotrophoblast Is a Barrier to Maternal-Fetal Transmission of Herpes Simplex Virus1. Biol. Reprod..

[95] Mimura N., Nagamatsu T., Morita K., Taguchi A., Toya T., Kumasawa K., Iriyama T., Kawana K., Inoue N., Fujii T. (2022). Suppression of Human Trophoblast Syncytialization by Human Cytomegalovirus Infection. Placenta.

[96] Kim C.J., Romero R., Chaemsaithong P., Kim J.-S. (2015). Chronic Inflammation of the Placenta: Definition, Classification, Pathogenesis, and Clinical Significance. Am. J. Obstet. Gynecol..

[97] Lindholm K., O’Keefe M. (2019). Placental Cytomegalovirus Infection. Arch. Pathol. Lab. Med..

[98] Pereira L., Petitt M., Tabata T. (2013). Cytomegalovirus Infection and Antibody Protection of the Developing Placenta. Clin. Infect. Dis..

[99] Hamilton S.T., Scott G., Naing Z., Iwasenko J., Hall B., Graf N., Arbuckle S., Craig M.E., Rawlinson W.D. (2012). Human Cytomegalovirus-Induces Cytokine Changes in the Placenta with Implications for Adverse Pregnancy Outcomes. PLoS ONE.

[100] Reiter R.J., Mayo J.C., Tan D., Sainz R.M., Alatorre-Jimenez M., Qin L. (2016). Melatonin as an Antioxidant: Under Promises but over Delivers. J. Pineal Res..

[101] Perrone S., Negro S., Tataranno M.L., Buonocore G. (2010). Oxidative Stress and Antioxidant Strategies in Newborns. J. Matern.-Fetal Neonatal Med..

[102] Jiménez-Osorio A.S., Carreón-Torres E., Correa-Solís E., Ángel-García J., Arias-Rico J., Jiménez-Garza O., Morales-Castillejos L., Díaz-Zuleta H.A., Baltazar-Tellez R.M., Sánchez-Padilla M.L. (2023). Inflammation and Oxidative Stress Induced by Obesity, Gestational Diabetes, and Preeclampsia in Pregnancy: Role of High-Density Lipoproteins as Vectors for Bioactive Compounds. Antioxidants.

[103] Saucedo R., Ortega-Camarillo C., Ferreira-Hermosillo A., Díaz-Velázquez M.F., Meixueiro-Calderón C., Valencia-Ortega J. (2023). Role of Oxidative Stress and Inflammation in Gestational Diabetes Mellitus. Antioxidants.

[104] Foka F.E.T., Mufhandu H.T. (2023). Current ARTs, Virologic Failure, and Implications for AIDS Management: A Systematic Review. Viruses.

[105] Lembo C., Buonocore G., Perrone S. (2021). Oxidative Stress in Preterm Newborns. Antioxidants.

[106] Perrone S., Manti S., Petrolini C., Dell’Orto V.G., Boscarino G., Ceccotti C., Bertini M., Buonocore G., Esposito S.M.R., Gitto E. (2023). Oxygen for the Newborn: Friend or Foe?. Children.

[107] Martin A., Faes C., Debevec T., Rytz C., Millet G., Pialoux V. (2018). Preterm Birth and Oxidative Stress: Effects of Acute Physical Exercise and Hypoxia Physiological Responses. Redox Biol..

[108] Capasso L., Vento G., Loddo C., Tirone C., Iavarone F., Raimondi F., Dani C., Fanos V. (2019). Oxidative Stress and Bronchopulmonary Dysplasia: Evidences From Microbiomics, Metabolomics, and Proteomics. Front. Pediatr..

[109] Cannavò L., Perrone S., Viola V., Marseglia L., Di Rosa G., Gitto E. (2021). Oxidative Stress and Respiratory Diseases in Preterm Newborns. Int. J. Mol. Sci..

[110] Millán I., Piñero-Ramos J.D., Lara I., Parra-Llorca A., Torres-Cuevas I., Vento M. (2018). Oxidative Stress in the Newborn Period: Useful Biomarkers in the Clinical Setting. Antioxidants.

[111] Lubrano C., Parisi F., Cetin I. (2024). Impact of Maternal Environment and Inflammation on Fetal Neurodevelopment. Antioxidants.

[112] Kneeland R.E., Fatemi S.H. (2013). Viral Infection, Inflammation and Schizophrenia. Prog. Neuropsychopharmacol. Biol. Psychiatry.

[113] Andescavage N.N., Limperopoulos C. (2021). Placental Abnormalities in Congenital Heart Disease. Transl. Pediatr..

[114] Dawood F.S., Kittikraisak W., Patel A., Rentz Hunt D., Suntarattiwong P., Wesley M.G., Thompson M.G., Soto G., Mundhada S., Arriola C.S. (2021). Incidence of Influenza during Pregnancy and Association with Pregnancy and Perinatal Outcomes in Three Middle-Income Countries: A Multisite Prospective Longitudinal Cohort Study. Lancet Infect. Dis..

[115] Malhotra A., Allison B.J., Castillo-Melendez M., Jenkin G., Polglase G.R., Miller S.L. (2019). Neonatal Morbidities of Fetal Growth Restriction: Pathophysiology and Impact. Front. Endocrinol..

[116] Newsome K., Alverson C.J., Williams J., McIntyre A.F., Fine A.D., Wasserman C., Lofy K.H., Acosta M., Louie J.K., Jones-Vessey K. (2019). Outcomes of Infants Born to Women with Influenza A(H1N1)Pdm09. Birth Defects Res..

[117] Wang R., Yan W., Du M., Tao L., Liu J. (2021). The Effect of Influenza Virus Infection on Pregnancy Outcomes: A Systematic Review and Meta-Analysis of Cohort Studies. Int. J. Infect. Dis..

[118] Allgäuer L., Cabungcal J.-H., Yzydorczyk C., Do K.Q., Dwir D. (2023). Low Protein-Induced Intrauterine Growth Restriction as a Risk Factor for Schizophrenia Phenotype in a Rat Model: Assessing the Role of Oxidative Stress and Neuroinflammation Interaction. Transl. Psychiatry.

[119] Di Martino D.D., Avagliano L., Ferrazzi E., Fusè F., Sterpi V., Parasiliti M., Stampalija T., Zullino S., Farina A., Bulfamante G.P. (2022). Hypertensive Disorders of Pregnancy and Fetal Growth Restriction: Clinical Characteristics and Placental Lesions and Possible Preventive Nutritional Targets. Nutrients.

[120] Liong S., Choy K.H.C., De Luca S.N., Liong F., Coward-Smith M., Oseghale O., Miles M.A., Vlahos R., Valant C., Nithianantharajah J. (2024). Brain Region-Specific Alterations in Gene Expression Trajectories in the Offspring Born from Influenza A Virus Infected Mice. Brain Behav. Immun..

[121] Hung T.-H., Skepper J.N., Burton G.J. (2001). In Vitro Ischemia-Reperfusion Injury in Term Human Placenta as a Model for Oxidative Stress in Pathological Pregnancies. Am. J. Pathol..

[122] Lanzieri T.M., Leung J., Caviness A.C., Chung W., Flores M., Blum P., Bialek S.R., Miller J.A., Vinson S.S., Turcich M.R. (2017). Long-Term Outcomes of Children with Symptomatic Congenital Cytomegalovirus Disease. J. Perinatol..

[123] Fisher S., Genbacev O., Maidji E., Pereira L. (2000). Human Cytomegalovirus Infection of Placental Cytotrophoblasts In Vitro and In Utero: Implications for Transmission and Pathogenesis. J. Virol..

[124] Njue A., Coyne C., Margulis A.V., Wang D., Marks M.A., Russell K., Das R., Sinha A. (2020). The Role of Congenital Cytomegalovirus Infection in Adverse Birth Outcomes: A Review of the Potential Mechanisms. Viruses.

[125] Fasoulakis Z., Koutras A., Antsaklis P., Theodora M., Valsamaki A., Daskalakis G., Kontomanolis E.N. (2023). Intrauterine Growth Restriction Due to Gestational Diabetes: From Pathophysiology to Diagnosis and Management. Medicina.

[126] Das A. (2014). Cytomegalovirus-Induced Hepatitis in an Immunocompetent Patient. Am. J. Case Rep..

[127] Müller L., Di Benedetto S. (2024). Immunosenescence and Cytomegalovirus: Exploring Their Connection in the Context of Aging, Health, and Disease. Int. J. Mol. Sci..

[128] Hernández S., Catalán-García M., Morén C., García-Otero L., López M., Guitart-Mampel M., Milisenda J., Coll O., Cardellach F., Gratacós E. (2017). Placental Mitochondrial Toxicity, Oxidative Stress, Apoptosis, and Adverse Perinatal Outcomes in HIV Pregnancies Under Antiretroviral Treatment Containing Zidovudine. JAIDS J. Acquir. Immune Defic. Syndr..

[129] Elias A., Nelson B., Oputiri D., Geoffrey O.-B.P. (2013). Antiretroviral Toxicity and Oxidative Stress. Am. J. Pharmacol. Toxicol..

[130] Greer L.G., Sheffield J.S., Rogers V.L., Roberts S.W., McIntire D.D., Wendel G.D. (2010). Maternal and Neonatal Outcomes After Antepartum Treatment of Influenza With Antiviral Medications. Obstet. Gynecol..

[131] Kadambari S., Griffiths P.D., Sharland M. (2013). The Novel Antiviral Pipeline to Treat Severe Neonatal Viral Infections. Pediatr. Infect. Dis. J..

[132] Niu Y., Herrera E.A., Evans R.D., Giussani D.A. (2013). Antioxidant Treatment Improves Neonatal Survival and Prevents Impaired Cardiac Function at Adulthood Following Neonatal Glucocorticoid Therapy. J. Physiol..

[133] Perrone S., Laschi E., Buonocore G. (2020). Oxidative Stress Biomarkers in the Perinatal Period: Diagnostic and Prognostic Value. Semin. Fetal Neonatal Med..

[134] Peña-Bautista C., Durand T., Vigor C., Oger C., Galano J.-M., Cháfer-Pericás C. (2019). Non-Invasive Assessment of Oxidative Stress in Preterm Infants. Free Radic. Biol. Med..

[135] Longini M., Belvisi E., Proietti F., Bazzini F., Buonocore G., Perrone S. (2017). Oxidative Stress Biomarkers: Establishment of Reference Values for Isoprostanes, AOPP, and NPBI in Cord Blood. Mediators Inflamm..

[136] Perrone S., Laschi E., Buonocore G. (2019). Biomarkers of Oxidative Stress in the Fetus and in the Newborn. Free Radic. Biol. Med..

[137] Pranty A.I., Shumka S., Adjaye J. (2022). Bilirubin-Induced Neurological Damage: Current and Emerging IPSC-Derived Brain Organoid Models. Cells.

[138] Jayanti S., Ghersi-Egea J.-F., Strazielle N., Tiribelli C., Gazzin S. (2021). Severe Neonatal Hyperbilirubinemia and the Brain: The Old but Still Evolving Story. Pediatr. Med..

[139] Teng M., Wu T.-J., Jing X., Day B.W., Pritchard K.A., Naylor S., Teng R.-J. (2024). Temporal Dynamics of Oxidative Stress and Inflammation in Bronchopulmonary Dysplasia. Int. J. Mol. Sci..

[140] Martini S., Castellini L., Parladori R., Paoletti V., Aceti A., Corvaglia L. (2021). Free Radicals and Neonatal Brain Injury: From Underlying Pathophysiology to Antioxidant Treatment Perspectives. Antioxidants.

[141] Tenório M.C.d.S., Graciliano N.G., Moura F.A., Oliveira A.C.M.d., Goulart M.O.F. (2021). N-Acetylcysteine (NAC): Impacts on Human Health. Antioxidants.

[142] Shahin A.Y., Hassanin I.M.A., Ismail A.M., Kruessel J.S., Hirchenhain J. (2009). Effect of Oral N-Acetyl Cysteine on Recurrent Preterm Labor Following Treatment for Bacterial Vaginosis. Int. J. Gynecol. Obstet..

[143] Buhimschi C.S., Bahtiyar M.O., Zhao G., Abdelghany O., Schneider L., Razeq S.A., Dulay A.T., Lipkind H.S., Mieth S., Rogers L. (2021). Antenatal N-Acetylcysteine to Improve Outcomes of Premature Infants with Intra-Amniotic Infection and Inflammation (Triple I): Randomized Clinical Trial. Pediatr. Res..

[144] Joó J.G., Sulyok E., Bódis J., Kornya L. (2023). Disrupted Balance of the Oxidant–Antioxidant System in the Pathophysiology of Female Reproduction: Oxidative Stress and Adverse Pregnancy Outcomes. Curr. Issues Mol. Biol..

[145] DeFreitas M.J., Katsoufis C.P., Benny M., Young K., Kulandavelu S., Ahn H., Sfakianaki A., Abitbol C.L. (2022). Educational Review: The Impact of Perinatal Oxidative Stress on the Developing Kidney. Front. Pediatr..

[146] Poston L., Briley A., Seed P., Kelly F., Shennan A. (2006). Vitamin C and Vitamin E in Pregnant Women at Risk for Pre-Eclampsia (VIP Trial): Randomised Placebo-Controlled Trial. Lancet.

[147] McCance D.R., Holmes V.A., Maresh M.J., Patterson C.C., Walker J.D., Pearson D.W., Young I.S. (2010). Vitamins C and E for Prevention of Pre-Eclampsia in Women with Type 1 Diabetes (DAPIT): A Randomised Placebo-Controlled Trial. Lancet.

[148] Nüsken E., Voggel J., Saschin L., Weber L.T., Dötsch J., Alcazar M.A.A., Nüsken K.D. (2024). Kidney Lipid Metabolism: Impact on Pediatric Kidney Diseases and Modulation by Early-Life Nutrition. Pediatr. Nephrol..

[149] Di Fabrizio C., Giorgione V., Khalil A., Murdoch C.E. (2022). Antioxidants in Pregnancy: Do We Really Need More Trials?. Antioxidants.

[150] Cottart C., Nivet-Antoine V., Laguillier-Morizot C., Beaudeux J. (2010). Resveratrol Bioavailability and Toxicity in Humans. Mol. Nutr. Food Res..

[151] Ding J., Kang Y., Fan Y., Chen Q. (2017). Efficacy of Resveratrol to Supplement Oral Nifedipine Treatment in Pregnancy-Induced Preeclampsia. Endocr. Connect..

[152] Darby J.R.T., Mohd Dollah M.H.B., Regnault T.R.H., Williams M.T., Morrison J.L. (2019). Systematic Review: Impact of Resveratrol Exposure during Pregnancy on Maternal and Fetal Outcomes in Animal Models of Human Pregnancy Complications—Are We Ready for the Clinic?. Pharmacol. Res..

[153] Ramli I., Posadino A.M., Giordo R., Fenu G., Fardoun M., Iratni R., Eid A.H., Zayed H., Pintus G. (2023). Effect of Resveratrol on Pregnancy, Prenatal Complications and Pregnancy-Associated Structure Alterations. Antioxidants.

[154] Hannan N.J., Binder N.K., Beard S., Nguyen T.-V., Kaitu’u-Lino T.J., Tong S. (2018). Melatonin Enhances Antioxidant Molecules in the Placenta, Reduces Secretion of Soluble Fms-like Tyrosine Kinase 1 (SFLT) from Primary Trophoblast but Does Not Rescue Endothelial Dysfunction: An Evaluation of Its Potential to Treat Preeclampsia. PLoS ONE.

[155] Richter H.G., Hansell J.A., Raut S., Giussani D.A. (2009). Melatonin Improves Placental Efficiency and Birth Weight and Increases the Placental Expression of Antioxidant Enzymes in Undernourished Pregnancy. J. Pineal Res..

[156] Milczarek R., Hallmann A., Sokołowska E., Kaletha K., Klimek J. (2010). Melatonin Enhances Antioxidant Action of α-Tocopherol and Ascorbate against NADPH- and Iron-Dependent Lipid Peroxidation in Human Placental Mitochondria. J. Pineal Res..

[157] Fantasia I., Bussolaro S., Stampalija T., Rolnik D.L. (2022). The Role of Melatonin in Pregnancies Complicated by Placental Insufficiency: A Systematic Review. Eur. J. Obstet. Gynecol. Reprod. Biol..

[158] Naemi M., Farahani Z., Norooznezhad A.H., Khodarahmi R., Hantoushzadeh S., Ahangari R., Shariat M. (2021). Possible Potentials of Curcumin for Pregnancies Complicated by Intra-Uterine Growth Restriction: Role of Inflammation, Angiogenesis, and Oxidative Stress. Heliyon.

[159] Filardi T., Varì R., Ferretti E., Zicari A., Morano S., Santangelo C. (2020). Curcumin: Could This Compound Be Useful in Pregnancy and Pregnancy-Related Complications?. Nutrients.

[160] Tossetta G., Fantone S., Giannubilo S.R., Marzioni D. (2021). The Multifaced Actions of Curcumin in Pregnancy Outcome. Antioxidants.

[161] Epstein J., Sanderson I.R., MacDonald T.T. (2010). Curcumin as a Therapeutic Agent: The Evidence from in Vitro, Animal and Human Studies. Br. J. Nutr..

[162] Oyewole A.O., Birch-Machin M.A. (2015). Mitochondria-targeted Antioxidants. FASEB J..

[163] Yang Y., Xu P., Zhu F., Liao J., Wu Y., Hu M., Fu H., Qiao J., Lin L., Huang B. (2021). The Potent Antioxidant MitoQ Protects Against Preeclampsia During Late Gestation but Increases the Risk of Preeclampsia When Administered in Early Pregnancy. Antioxid. Redox Signal.

[164] Feniouk B.A., Skulachev V.P. (2017). Cellular and Molecular Mechanisms of Action of Mitochondria-Targeted Antioxidants. Curr. Aging Sci..

[165] Jiang Q., Yin J., Chen J., Ma X., Wu M., Liu G., Yao K., Tan B., Yin Y. (2020). Mitochondria-Targeted Antioxidants: A Step towards Disease Treatment. Oxid. Med. Cell. Longev..

[166] Barletta M.A., Marino G., Spagnolo B., Bianchi F.P., Falappone P.C.F., Spagnolo L., Gatti P. (2022). Coenzyme Q10 + Alpha Lipoic Acid for Chronic COVID Syndrome. Clin. Exp. Med..

[167] Mathys L., Balzarini J. (2016). The Role of Cellular Oxidoreductases in Viral Entry and Virus Infection-Associated Oxidative Stress: Potential Therapeutic Applications. Expert. Opin. Ther. Targets.

[168] Daskou M., Fotooh Abadi L., Gain C., Wong M., Sharma E., Kombe Kombe A.J., Nanduri R., Kelesidis T. (2023). The Role of the NRF2 Pathway in the Pathogenesis of Viral Respiratory Infections. Pathogens.

[169] Kesic M.J., Simmons S.O., Bauer R., Jaspers I. (2011). Nrf2 Expression Modifies Influenza A Entry and Replication in Nasal Epithelial Cells. Free Radic. Biol. Med..

[170] McCord J.M., Hybertson B.M., Cota-Gomez A., Geraci K.P., Gao B. (2020). Nrf2 Activator PB125^®^ as a Potential Therapeutic Agent against COVID-19. Antioxidants.

[171] Marrocco I., Altieri F., Peluso I. (2017). Measurement and Clinical Significance of Biomarkers of Oxidative Stress in Humans. Oxid. Med. Cell. Longev..

[172] Zhang H., Lin J., Zhao H. (2024). Impacts of Maternal Preeclampsia Exposure on Offspring Neuronal Development: Recent Insights and Interventional Approaches. Int. J. Mol. Sci..

[173] Meral G., Aslan E.S., Burkay N., Alper Acar E.G., Karagöz M.F., Özkaya M., Sahin E., Alp M.Y. (2024). Importance of Using Epigenetic Nutrition and Supplements Based on Nutrigenetic Tests in Personalized Medicine. Cureus.

[174] Sakowicz A., Bralewska M., Rybak-Krzyszkowska M., Grzesiak M., Pietrucha T. (2023). New Ideas for the Prevention and Treatment of Preeclampsia and Their Molecular Inspirations. Int. J. Mol. Sci..

[175] Villanueva-Paz M., Morán L., López-Alcántara N., Freixo C., Andrade R.J., Lucena M.I., Cubero F.J. (2021). Oxidative Stress in Drug-Induced Liver Injury (DILI): From Mechanisms to Biomarkers for Use in Clinical Practice. Antioxidants.

[176] Battista C., Howell B.A., Siler S.Q., Watkins P.B. (2018). An Introduction to DILIsym^®^ Software, a Mechanistic Mathematical Representation of Drug-Induced Liver Injury. Drug-Induced Liver Toxicity.

[177] Donato M.T., Tolosa L. (2021). High-Content Screening for the Detection of Drug-Induced Oxidative Stress in Liver Cells. Antioxidants.

[178] Silva L.L., Silvola R.M., Haas D.M., Quinney S.K. (2022). Physiologically Based Pharmacokinetic Modelling in Pregnancy: Model Reproducibility and External Validation. Br. J. Clin. Pharmacol..

[179] Thépaut E., Brochot C., Chardon K., Personne S., Zeman F.A. (2023). Pregnancy-PBPK Models: How Are Biochemical and Physiological Processes Integrated?. Comput. Toxicol..

[180] Dmitriev A.V., Rudik A.V., Karasev D.A., Pogodin P.V., Lagunin A.A., Filimonov D.A., Poroikov V.V. (2021). In Silico Prediction of Drug–Drug Interactions Mediated by Cytochrome P450 Isoforms. Pharmaceutics.

[181] Wu F., Zhou Y., Li L., Shen X., Chen G., Wang X., Liang X., Tan M., Huang Z. (2020). Computational Approaches in Preclinical Studies on Drug Discovery and Development. Front. Chem..

